# Task-specific regional circuit adaptations in distinct mouse retinal ganglion cells

**DOI:** 10.1126/sciadv.adp7075

**Published:** 2025-04-23

**Authors:** Jonathan Oesterle, Yanli Ran, Paul Stahr, Jason N. D. Kerr, Timm Schubert, Philipp Berens, Thomas Euler

**Affiliations:** ^1^ Institute for Ophthalmic Research, University of Tübingen, Tübingen, Germany.; ^2^ Werner Reichardt Centre for Integrative Neuroscience, University of Tübingen, Tübingen, Germany.; ^3^ Hertie Institute for AI in Brain Health, University of Tübingen, Tübingen, Germany.; ^4^ Institute of Physiology, School of Basic Medical Sciences, Lanzhou University, Lanzhou, Gansu, China.; ^5^ Max Planck Institute for Neurobiology of Behavior, Bonn, Germany.; ^6^ Tübingen AI Center, University of Tübingen, Tübingen, Germany.

## Abstract

In the mouse retina, sustained ON alpha (sONα) retinal ganglion cells (RGCs) have different dendritic and receptive field sizes along the nasotemporal axis, with temporal sONα RGCs likely playing a role in visually guided hunting. Thus, we hypothesized that this cell type also exhibits regional adaptations in dendritic signal processing and that these adaptations are advantageous for prey capture. Here, we measured dendritic signals from individual sONα RGCs at different retinal locations. We measured both postsynaptic Ca^2+^ signals at dendrites and presynaptic glutamate signals from bipolar cells (BCs). We found that temporal sONα RGCs exhibit, in addition to sustained-ON signals with only weak surrounds, signals with strong surround suppression, which were not present in nasal sONα RGCs. This difference was also present in the presynaptic inputs from BCs. Last, using population models in an encoder-decoder paradigm, we showed that these adaptations might be beneficial for detecting crickets in hunting behavior.

## Teaser

The mouse retina shows regional adaptations in signal processing that enhance their ability to detect prey during hunting.

## INTRODUCTION

The architecture of the visual system of animals is shaped by the statistics of the environment as well as behavioral demands (*1*). Thus, although their retina is based on a common blueprint, vertebrates show substantial variations in retinal architecture, including many regional adaptations within the retina. This underscores the influence of evolutionary pressures and ecological niches on visual systems (*1*, *2*).

Some species, like many primates and certain birds, have developed foveae, that is, regional specializations for high-acuity vision with distinct architecture compared to the peripheral retina (*3*). However, also, non-foveated species typically feature local specializations of their retinas: For instance, zebrafish have a region of higher retinal ganglion cell (RGC) density, also referred to as the “strike zone,” which contains many ultraviolet (UV)–sensitive photoreceptors and is believed to play a crucial role in hunting (*4*–*6*). Similarly, in mice, regional adaptations can already be found at the photoreceptor layer. For example, in some species of the genus *Mus*, including steppe mice (*Mus spicilegus*) and also the derivative lab strain C57BL/6J, short- (S-) and medium (M-) wavelength–sensitive opsin expression follows a pronounced gradient along the dorso-ventral axis (*7*–*10*), resulting in a green-sensitive dorsal retina and a UV-sensitive “hotspot” in the naso-ventral retina (*11*). These spectral sensitivity differences are propagated via the BCs (*12*, *13*) to the RGCs (*14*). Other mouse species from distinct habitats, such as the wood mice (*Apodemus sylvaticus*) that—in contrast to steppe mice—are typically found in forests (*15*), completely lack this gradient (*7*). At the level of RGCs, mice have also been shown to exhibit a region of lower RGC density in the dorsal retina (*16*, *17*) and several regional adaptations that are specific to distinct RGC types (*18*, *19*).

Here, we focused on sustained ON alpha (sONα) RGCs [EyeWire: 8w; (*20*, *21*)], which have been shown to vary across space at the level of their morphology: Temporal sONα have much smaller dendritic arbors and exhibit a higher cell density compared to nasal cells (*17*, *22*, *23*). Notably, temporal sONα RGCs have also been linked to visually guided hunting, suggestive of a direct connection between their morphology and functional significance (*24*, *25*).

To better understand whether these cells also display adaptations on the functional level and how these arise from their dendritic input and cellular computations, we recorded dendritic Ca^2+^ signals and excitatory synaptic inputs to sONα cells in different regions of the retina. On the basis of morphological and functional data, we then created computational population models of both nasal and temporal sONα RGCs. We used these models to encode the visual scene as seen by freely moving mice hunting crickets (*25*). We trained a decoder to estimate the presence of a cricket from the population responses in a binary classification task. We found that the decoder performed much better for temporal sONα RGCs compared to nasal ones. Moreover, our simulation indicated that stronger surround inhibition already at the level of presynaptic neurons was likely the cellular mechanism responsible for the better performance of temporal sONα RGCs in this task. Together, our results suggest that regional changes in presynaptic circuits and dendritic signal integration are key mechanisms in tuning temporal sONα RGCs for detecting small objects such as insects.

## RESULTS

### Recording sONα RGCs across the retina

**Fig. 1.**
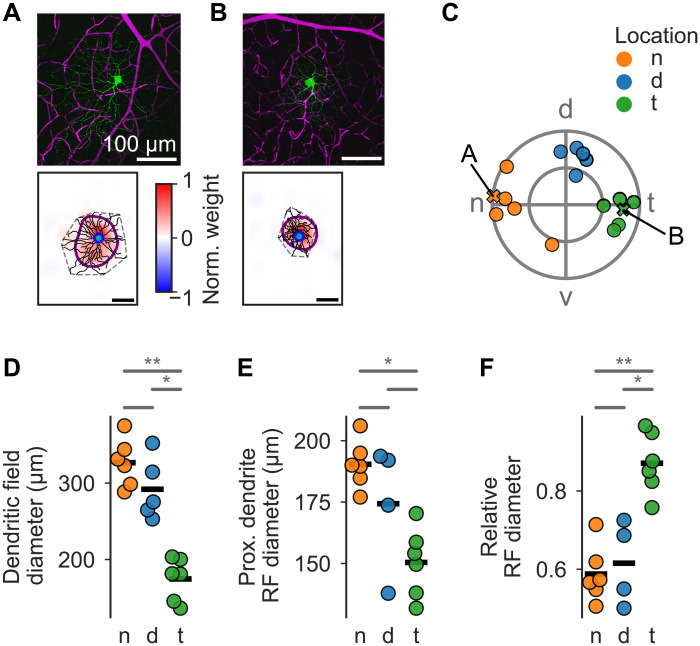
Nasal and temporal sONα cells differ in dendritic arbor and somatic RF size. (**A**) Top, two-photon image of dye-injected nasal sONα RGC [green, OGB-1; magenta, sulforhodamine 101 (SR-101); weighted two-channel z projection]. Bottom, dendritic skeleton reconstructed from z stack, its convex hull (gray dashed) overlaid with the RF (color map) estimated from a Ca^2+^ signal in a proximal dendrite ROI [blue (see Materials and Methods)], and the respective RF outline estimate (purple ellipse). (**B**) As in (A) but for a temporal cell. Scale bars, (A) and (B) 100 μm. (**C**) Retinal cell locations of all sONα RGCs from which dendritic Ca^2+^ signals were recorded (n, nasal, orange; d, dorsal, blue; t, temporal, green). RGCs in (A) and (B) are highlighted. The outer circle indicates the edge of the retina (see Materials and Methods). (**D** to **F**) Statistical comparison of cells in (C) using Kruskal-Wallis and Dunn’s tests with Benjamini-Hochberg correction, *P < 0.05, **P < 0.01. Data means are shown as black bars. (D) Dendritic field diameter estimated from convex hull (see Materials and Methods). (E) Proximal dendrite RF diameter if it was recorded. (F) Proximal dendrite RF diameter divided by dendritic field diameter.

**Fig. 2.**
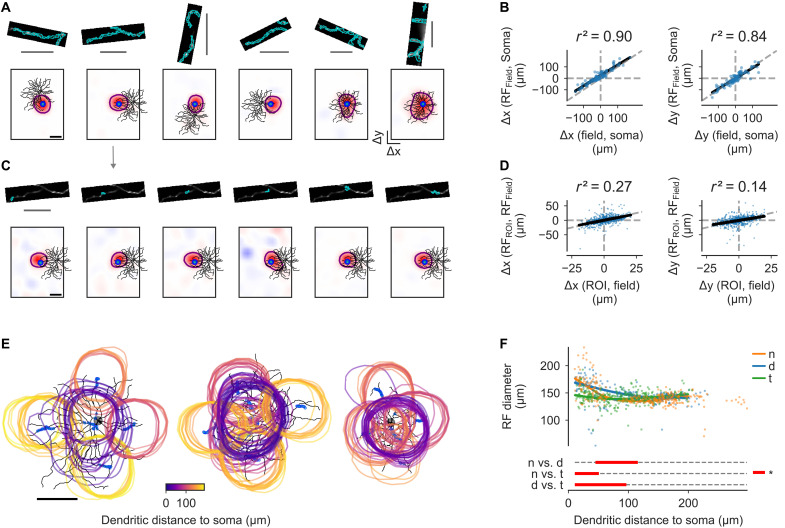
Dendritic RFs of sONα cells differ between retinal locations. (**A**) Morphology of RGC in Fig. 1A, overlaid with ROIs and respective RFs. Top row, Ca^2+^ signal of dendritic recording fields averaged over time with highlighted ROIs (blue). Bottom row, morphology (black) and location of respective recording fields (blue) overlaid with outline (purple) of field RF (see Materials and Methods). (**B**) Linear regression and Pearson’s *r*^2^ for field RF center positions w.r.t. soma versus field center positions w.r.t. to soma for x (left) and y (right), respectively. (**C**) Top row, as in (A) but for RFs from individual ROIs from the second field in (A). Bottom row, as in (A) but for the respective ROI RFs. (**D**) Linear regression and Pearson’s *r*^2^ for ROI RF center w.r.t. Field RF center versus ROI center w.r.t. field center for x (left) and y (right), respectively. (**E**) All ROIs and ROI RF outlines after quality filtering (RF outlines color code ROI dendritic distance to soma), for an n cell, a d cell, and a t cell, respectively. (**F**) Diameters of ROI RF outline estimate (see Materials and Methods) as a function of ROI dendritic distance to soma by retinal region (color) and fits from a Generalized Additive Model [GAM (see Materials and Methods)]. Distances of significant difference between the groups are highlighted (bottom, red). Black and gray scale bars, [(A), (C), and (E)] 100 and 30 μm, respectively.

To analyze regional adaptations in dendritic signal processing of sONα RGCs, we recorded dendritic Ca^2+^ signals in response to visual stimulation of individual RGCs in the ex vivo, whole-mount mouse retina using two-photon imaging. For this, we injected individual RGCs with the fluorescent Ca^2+^ indicator dye Oregon Green BAPTA-1 (OGB-1) using sharp electrodes (see Materials and Methods), resulting in labeling of individual cells (Fig. 1, A and B). After the functional recordings, we three-dimensionally (3D) reconstructed the respective RGCs (Figs. 1 and 2 and fig. S1) and mapped the dendritic recording field onto the morphology (see Materials and Methods). We grouped the recorded sONα RGCs into three groups based on their retinal location: nasal (n), dorsal (d), and temporal (t) (Fig. 1C). Note that we included the ventral cell in the n group because its morphology and functional properties matched this group. We estimated receptive fields (RFs) from a binary dense noise stimulus (20 by 15 pixels, 30 μm per pixel) that was centered on the respective recording fields. As we focused on dendritic recordings, we initially did not record from RGC somata but, for most cells, from dendrites very close to the soma. These RFs can be used as a proxy for somatic RFs (figs. S4 and S5).

Consistent with previous reports (*22*), we found that, compared to nasal or dorsal cells, temporal sONα RGCs had both smaller dendritic fields (n versus d: *P* = 0.34; d versus t: *P* = 0.036; n versus t: *P* = 0.0022; Fig. 1D), and smaller (soma-like) RFs (n versus d: *P* = 0.34; d versus t: *P* = 0.16; n versus t: *P* = 0.013; Fig. 1E), but larger relative RF sizes, i.e., the RF size divided by the dendritic field size (n versus d: *P* = 0.89; d versus t: *P* = 0.018; n versus t: *P* = 0.0089; Fig. 1F). Note that in Fig. 1E, the difference between dorsal and temporal was not significant, likely because of the smaller sample size and one outlier RF in the d group (fig. S5).

### Dendritic signals reflect localized input processing

Next, we measured Ca^2+^ signals across the dendritic arbor of individual cells. For each dendritic recording field (64 by 16 pixels, 31.25 Hz), we extracted regions of interest (ROIs) using local pixel correlations (see Materials and Methods). We estimated an RF using the aforementioned dense noise stimulus for each ROI and additionally for each field by combining the respective ROIs in each field (see Materials and Methods). The RF centers followed the location of the dendritic recording fields across the dendritic trees (Fig. 2, A and B). Even within fields, the relative RF center positions [with respect to (w.r.t.) the field RF] were correlated with the relative position of the individual ROIs (w.r.t. the field center) (Fig. 2, C to E), suggesting that the recorded signals were electrically isolated dendritic signals and not mainly back-propagated somatic signals.

In dendrites close to the soma, n and d cells had larger RFs than t cells, with no significant difference between n and d cells (Fig. 2, E and F). However, for more distal dendrites (≥116 μm), there were no significant differences between the retinal locations (Fig. 2, E and F). This suggests that n and t cells integrate dendritic signals differently at proximal dendrites and presumably also the soma. The d cells had significantly larger RFs for intermediate distances between approximately 50 and 100 μm than both t and n cells.

### Dendritic signals have diverse spatial and temporal response properties

**Fig. 3.**
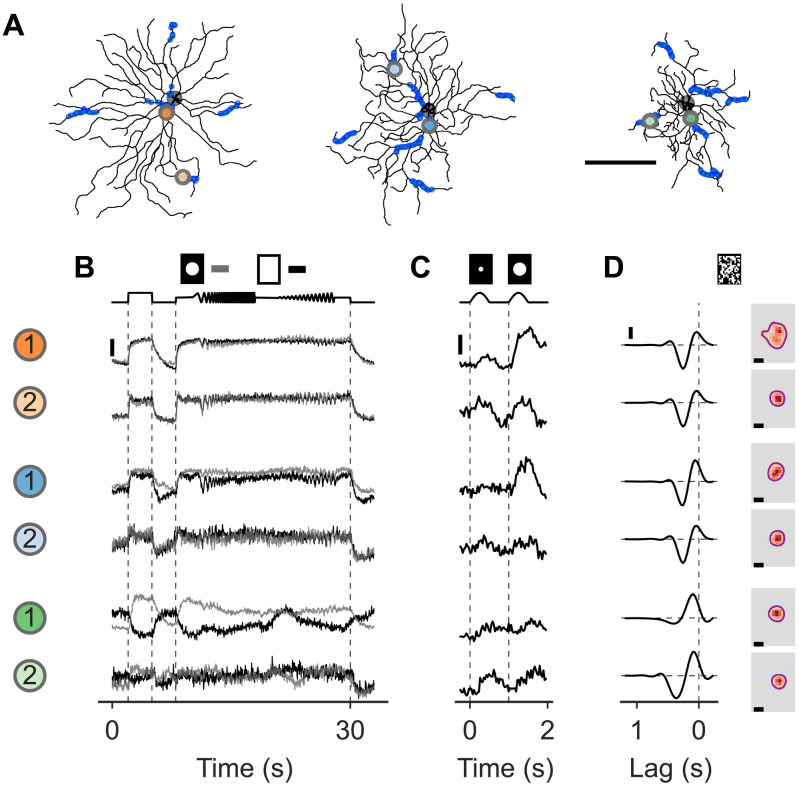
Dendritic signals have diverse spatial and temporal response properties. (**A**) Cells from Fig. 2E with two example ROIs highlighted for each cell (scale bar, 100 μm). (B to D) Responses of example ROIs to all light stimuli used. (**B**) Chirp (local, gray; global, black) response averages over repetitions [scale bar, a response amplitude of 2 (a.u.)]. (**C**) Sine-spot response averages [scale bar, response amplitude of 2 (a.u.)]. (**D**) Dense-noise response shown as temporal [left, vertical scale bar, response amplitude of 0.5 (a.u.)] and spatial (right, scale bar, 100 μm) RFs.

To analyze temporal and spatial properties of dendritic signal integration, we used, in addition to the noise stimulus, a local (300-μm diameter) and a global (≈800-μm diameter) “chirp” stimulus, and, because of limited recording time only for some fields, a “sine-spot” stimulus consisting of a small (60-μm diameter) and medium spot (300 μm) played in alternation (Fig. 3) (see Materials and Methods). As for the noise, the stimuli were always centered on the recording site. For the chirp stimulus, we found that in some cases, responses to the local and global chirp were almost identical (Fig. 3, A and B). However, in other cases, only the local chirp stimulus resulted in an “ON” response while the global chirp resulted in an “ON-suppressed” response, likely because of a stronger surround stimulation (Fig. 3B). In many cases, even the 60-μm spot of the sine-spot stimulus was able to reliably evoke responses (Fig. 3C), sometimes stronger than the 300-μm spot, indicating first, that these postsynaptic dendritic signals are sensitive to localized, small light spots, and second, that the ON component of these signals is likely dominated by excitatory inputs from only a few BCs close to the respective ROIs’ locations and activating more BCs more distant does not increase the response. We also found that the RFs of different ROIs did not only vary in their spatial but also their temporal tuning, ranging from transient biphasic temporal RF to more sustained monophasic temporal RFs (Fig. 3D).

### sONα RGCs exhibit regional differences in dendritic signal integration

**Fig. 4.**
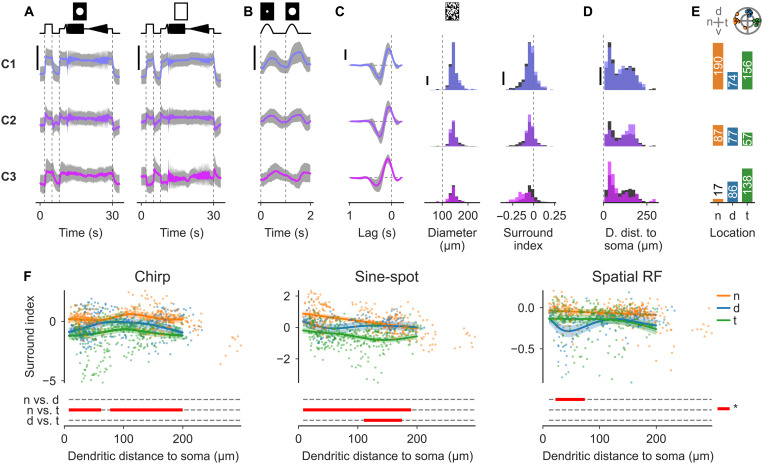
Temporal sONα show postsynaptic dendritic signals with strong surround suppression. Clustered postsynaptic Ca^2+^ signals of all ROIs exceeding the quality threshold. Responses were clustered on the basis of the local and global chirp responses. All traces used for clustering and a respective dendrogram are shown in fig. S6A. Data are split by the three Ca^2+^ clusters C1 (top), C2 (middle), and C3 (bottom). (**A**) Local (left) and global (right) chirp responses [scale bars, a response amplitude of 2 (a.u.)]. (**B**) As in (A) but for sine-spot responses [scale bar, response amplitude of 2 (a.u.)]. (**C**) RF properties: temporal RF [left, scale bar, response amplitude of 0.5 (a.u.)], spatial RF diameter (middle), and surround index (right). [(A) to (C)] All traces are shown as cluster means ± 1 SD. (**D**) ROI distribution of dendritic distance to soma. [(C) and (D)] For all histograms, the distribution across all clusters, scaled to cover the same area, is shown in the background (gray), and scale bars, 30 ROIs. (**E**) ROI counts per regional group. Top, cell locations for reference; the outer circle indicates the edge of the retina (see Materials and Methods). (**F**) Surround index as a function of dendritic distance to soma by retinal region (color) and fits from a GAM (see Materials and Methods). Distances of significant difference between the groups are highlighted (bottom, red). A surround index (see Materials and Methods) was computed from the local and global chirp stimulus (left), the sine-spot stimulus (middle), and from the spatial RF (right).

Overall, we found that the dendritic Ca^2+^ signals in response to the chirp stimuli were quite diverse, especially for the global chirp where some of the ROIs showed ON-suppressed responses (Fig. 3B and fig. S6A). This dendritic signal diversity could arise from differences in local synaptic inputs and/or electrical anatomy at the ROI (e.g. the distance to the soma). To distinguish between these possibilities, we summarized the most prominent signal features by clustering the ROIs based on their local and global chirp responses using a hierarchical clustering algorithm (see Materials and Methods). On the basis of the dendrogram, we selected a distance threshold to strike a balance between simplifying the data and not merging very different responses, resulting in three clusters (fig. S6A). The three clusters mostly differed in their transience and their strength of the surround suppression and could be described as ON-sustained (C1), ON-weakly-transient (C2), and “local-ON–global-ON–suppressed” (C3) (Fig. 4).

The ON-sustained cluster C1 (420 ROIs; Fig. 4, top row) showed a highly sustained response and only very weak surround suppression. The RF diameters of this cluster ranged from the smallest (≤100 μm) to the largest values we observed (>250 μm). The responses of the ON-weakly-transient cluster C2 (221 ROIs; Fig. 4, middle row) also showed little surround suppression and a strong sustained component, but it was more transient than C1. Compared to the other clusters, it was found more often in distal dendrites. The local-ON–global-ON–suppressed cluster C3 (241 ROIs; Fig. 4, bottom row) only showed a sustained ON response to the local chirp, while for the global chirp, the response was ON-suppressed, indicating a strong surround suppression (Fig. 4A) that was also visible in the sine-spot (Fig. 4B) and dense noise responses (Fig. 4C). Many ROIs of this cluster were located at an intermediate distance to the soma (59% of C3 ROIs between 25 and 75 μm; C2: 32%; C1 43%) and only a few very close to it (5% of C3 ROIs closer than 25 μm; C2: 10%; C1: 12%).

Next, we analyzed the contribution of cells and their retinal regions to the three clusters (Fig. 4E). We found that ROIs of n cells were almost exclusively found in clusters C1 (65%) and C2 (30%), whereas ROIs of d cells were relatively evenly distributed (C1: 31%; C2: 32%; C3: 36%) and ROIs of t cells where mostly found in C1 (44%) and C3 (39%). Together, this suggests a difference in transience and surround suppression in dendritic signals between nasal, dorsal, and temporal circuits.

To analyze the spatial distribution of signal transience and surround suppression, we computed a transience index from the local chirp responses and the temporal RF kernels (fig. S7A), and surround indexes from the chirps, the sine-spot, and the spatial RFs as a function of dendritic distance to soma (Fig. 4F). We found that for the local chirp, transience was not significantly different between retinal regions, whereas for the temporal RF kernels, transience was lower in t compared to n cells with significant differences in very proximal and also distal dendrites (fig. S7B). However, the most notable difference we found was the stronger surround suppression in t versus n cells; the surround suppression was stronger for all stimuli, with significant differences for the chirps and the sine-spot along almost the whole dendrite.

**Fig. 5.**
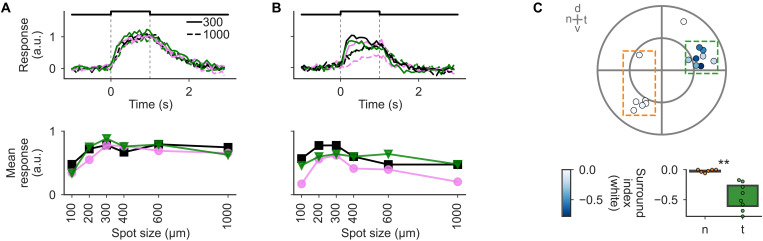
Temporal sONα have stronger somatic surround suppression in the temporal compared to the nasal retina. (**A**) Top, normalized somatic spot response of example nasal cell to spots with different diameters, 300 μm (solid lines) and 1000 μm (dashed lines), and different wavelengths, green (green lines), UV (violet lines), and white (black lines), as mean over repetitions. Bottom, mean responses as the area under the curve as a function of spot sizes. (**B**) As in (A) but for example temporal cell. (**C**) Top, surround index (SI) (color coded), computed from the white spots, that compares the maximum response to the response of the largest spot (see Materials and Methods) as a function of retinal location. Boxes show quartiles. The inner and outer circles indicate a distance of 1 and 2 mm from the optic disk, respectively. Bottom, SI of n versus t cells (from dashed boxes in top) compared using Mann-Whitney *U* test, **P < 0.01.

This strong surround suppression in t cells was unexpected because previous studies on sONα cells reported only weak surround suppression on the somatic level for this cell type (*24*, *26*). However, they used a different stimulus type, namely a spot with various diameters and different light conditions (see Discussion). To see whether the strong surround suppression we observed on the dendritic level is also visible on the somatic level under our light conditions, we performed additional somatic recordings using a colored spot stimulus (Fig. 5 and fig. S8). Indeed, the somatic surround suppression followed the same retinal distribution as that in the dendrites: *n* cells (Fig. 5A) had a very weak surround, while t cells had a significantly stronger one (Fig. 5, B and C; *P* = 0.002).

### Nasal and temporal sONα RGCs receive different excitatory synaptic inputs

**Fig. 6.**
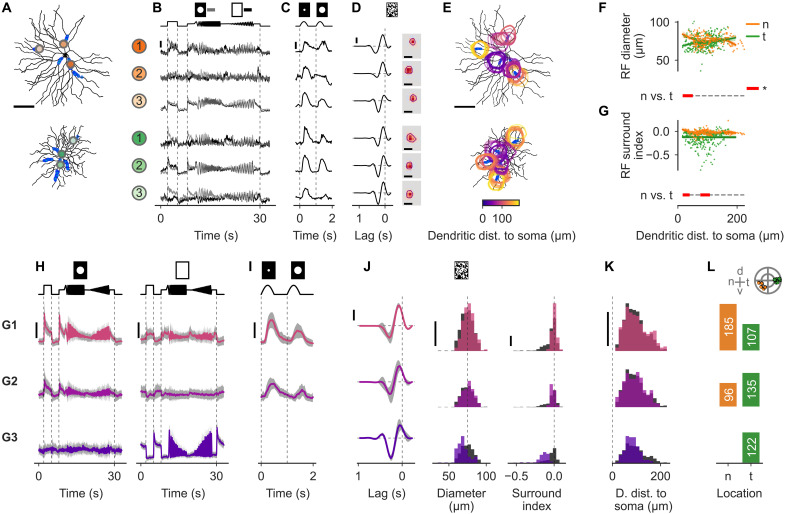
Strong surround suppression originates presynaptically in temporal sONα cells. (**A**) Cell morphologies (black) with three example ROIs highlighted for each cell. (**B** to **D**) Responses of example ROIs to all light stimuli used. (B) Chirp (local, gray; global, black) response averages over repetitions. (C) Sine-spot response averages. (D) Dense-noise responses are shown as temporal (left) and spatial (right) RFs. (**E**) Spatial RF outlines mapped on the morphologies (outlines color code ROI dendritic distance to soma). Horizontal scale bars, [(A), (D), and (E)] 100 μm. (**F**) RF diameter as a function of ROI dendritic distance to soma for nasal (n, orange) and temporal (t, green) cells, and fits from a GAM (see Materials and Methods). Distances of significant difference between n and t are highlighted (bottom, red). (**G**) As in (F) but for the RF surround index (see Materials and Methods). (**H** to **L**) As in Fig. 4 but for the clustered presynaptic glutamate signals of all ROIs exceeding the quality threshold. Responses were clustered on the basis of the local and global chirp responses. All traces used for clustering and a respective dendrogram are shown in fig. S6B. Data are split by the three glutamate clusters G1 (top), G2 (middle), and G3 (bottom). (H) Local (left) and global (right) chirp responses. (I) Sine-spot responses. Scale bars, [(B), (C), (H), and (I)] response amplitude of 2 (a.u.). (J) RF properties: temporal RF (left), spatial RF diameter (middle), and surround index (right). [(D) and (J)] Trace scale bars, response amplitude of 0.5 (a.u.). (K) ROI distribution of dendritic distance to soma. [(J) and (K)] Histogram scale bars, 30 ROIs. (L) ROI counts per regional group. Top, cell locations for reference; the outer circle indicate the edge of the retina (see Materials and Methods).

To investigate the origin of the postsynaptic surround suppression that was present in temporal (and dorsal), but not in nasal cells, we conducted a second set of experiments where we measured excitatory synaptic inputs onto the dendrites of sONα RGCs using the glutamate biosensor iGluSnFR (see Materials and Methods) and repeated the analysis from above for this dataset. Here, we restricted the cell locations to nasal (n) and temporal (t) for simplicity. We recorded signals across the dendrites (Fig. 6A) in response to the local and global chirp (Fig. 6B), the sine-spot stimulus (Fig. 6C), and the dense noise (Fig. 6D). As for the Ca^2+^ data, the RF centers were always close to the located recording sites (distance from ROI to RF center 31 ± 14 μm; mean ± 1 SD) (Fig. 6E). RF sizes were smaller in presynaptic compared to postsynaptic Ca^2+^ signals, likely because the postsynaptic dendritic signals are not only influenced by local inputs but also by inputs to adjacent dendritic segments and, to a lesser degree, by inputs to the whole dendritic branch. To compare the excitatory synaptic inputs of n and t sONα RGCs, we first looked at the RF sizes in relation to the ROIs’ distances to soma for both groups (Fig. 6F). We found that, close to the soma, RF sizes of nasal cells were slightly larger, while for larger distances, there was no significant difference between the two groups. Next, we quantified the strength of the antagonistic surround, measured as surround index (see Materials and Methods), in the RFs (Fig. 6G). The RF surround was stronger in t cells compared to n cells both close to the soma and for intermediate distances, suggesting that the surround suppression observed in t RGCs on the postsynaptic level (Fig. 4) may originate from synaptic glutamate input with stronger surround suppression. We also compared the transience between retinal regions and found that, as for the Ca^2+^ signals, there was no significant difference between the transience estimated from the local chirp responses (fig. S7, C and D). However, when comparing the temporal RFs, we found them to be less transient in n cells compared to t cells, especially close to the soma. This contrasts the more transient RFs of n cells we found in the postsynaptic signals. Only for the presynaptic signals, the transience indexes computed from the chirp and temporal RFs were anticorrelated (fig. S7. E and F), suggesting a complex interaction of transience and center-surround stimulation, as the chirp is spatially more uniform than the noise stimulus. This may also explain the difference between pre- and postsynaptic signals, as postsynaptic RFs are larger and integrate the signal from multiple inputs with potentially overlapping centers and surrounds of BCs.

To further compare the Ca^2+^ and glutamate signals, we clustered the glutamate signals using the same method as for the Ca^2+^, i.e., by clustering their local and global chirp responses (fig. S6B). Again, we found three clusters, one ON-sustained (G1) and two local-ON–global-ON–suppressed clusters with weak (G2) and strong (G3) suppression (Fig. 6, H to L).

The ON-sustained cluster (G1; Fig. 6, H to L, top row) had sustained ON responses for both chirp stimuli, with a preference for the smaller diameter. This cluster had the largest average RF size and the weakest RF surround suppression. The distance-to-soma distribution was relatively even, with little difference from the other clusters. The local-ON–global-ON–suppressed cluster with weak suppression (G2; Fig. 6, H to L, middle row) had a strong and sustained ON response for the local chirp. The global chirp response was ON-suppressed and more variable. The preference for the smaller spot was more prominent compared to cluster G1. RF sizes were slightly smaller and surround suppression slightly stronger than for G1. The local-ON–global-ON–suppressed cluster with strong suppression (G3; Fig. 6, H to L, bottom row) had a weak ON response for the local chirp. The response was completely suppressed by the global chirp. In G3, RF sizes were the smallest and RF surround suppression was the strongest among the three clusters.

Together, similar to the Ca^2+^ clusters (Fig. 4), we found that the glutamate clusters (Fig. 6, H to L) with stronger surround suppression were more frequently found in t cells, with ROIs from cluster G3 with the strongest suppression exclusively in t cells. This indicates that the stronger surround suppression observed in the dendritic Ca^2+^ signals of temporal sONα cells is at least partially inherited from the BCs inputs, likely reflecting presynaptic suppression. In contrast to Ca^2+^ signals, the glutamate RF kernels were more transient in t versus n cells, with nasal cells receiving less transient inputs close to the soma (fig. S7).

### Regional adaptations in temporal sONα RGCs are well suited for prey capture

The results so far showed that sONα RGCs feature distinct regional adaptations not only in morphology but also in postsynaptic signal processing and presynaptic inputs. This was most notable in the strong surround suppression we observed in sONα cells in the temporal periphery of the retina. Notably, this region coincides with the binocular area of the mouse’s visual field, which also has been proposed to play a critical role in visually guided hunting (*24*). Therefore, we hypothesized that the regional adaptations in dendritic input and signal processing of sONα RGCs are adaptations beneficial for tasks like prey capture.

**Fig. 7.**
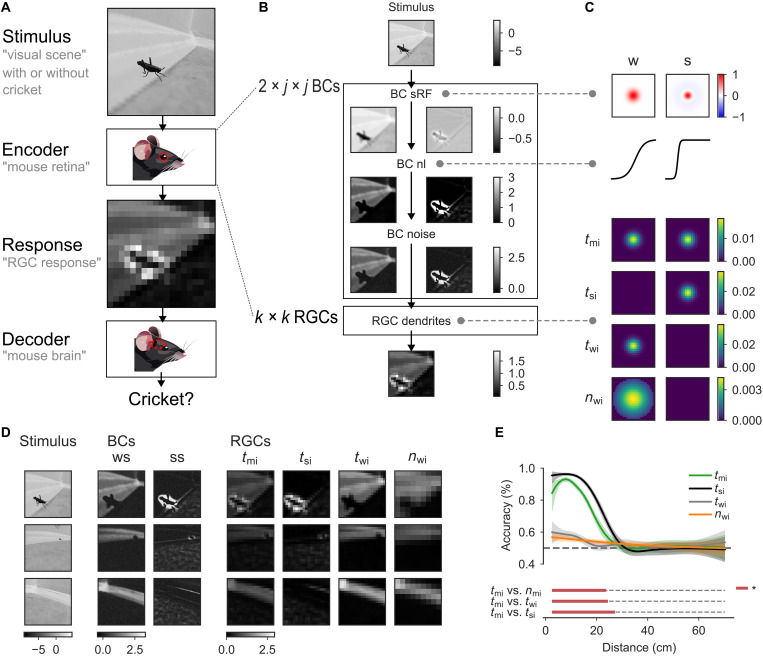
Regional adaptations in temporal sONα cells facilitate prey detection. (**A**) Encoder-decoder framework: A visual stimulus is presented to an encoder that simulates the response of an RGC population, which is fed to a decoder that has to estimate if the input contains a cricket or not. (**B**) Encoder structure and intermediate layer evaluations: The encoder, implemented as an artificial neuronal network, is modeled as two populations of *j* × *j* BCs and a population of *k* × *k* RGCs. Each BC population consists of a 2D convolution layer (BCs’ spatial RFs), followed by a nonlinearity, and a Gaussian additive noise layer. The RGC population consists of a single 3D convolutional layer (RGC dendrites), integrating the signals from the two BC populations. (**C**) Encoder parameter details. Top, the spatial RFs and the nonlinearities of the two BC populations, one with weak surround (w) and one with strong surround (s), respectively. Bottom, the dendritic weights of four different populations of RGCs: temporal (t) RGCs with inputs from either both (*t*_mi_), or only from w BCs (*t*_wi_), or s BCs (*t*_si_); and nasal (n) RGCs with inputs from w BCs only (*t*_wi_). (**D**) Simulated BC and RGC responses for three example stimuli, one with a close, one with a distal and one without a cricket. (**E**) Decoder accuracy fitted with a logistic GAM as a function of the cricket distance (see Materials and Methods), for the four RGC populations, each tested against the *t*_mi_ population.

To test this, we created an encoder-decoder paradigm (Fig. 7) (see Materials and Methods) with different sONα population models encoding scenes of a visually guided cricket hunt reconstructed in (*25*) and a decoder trained to detect the presence or absence of a cricket (Fig. 7A). This encoder-decoder paradigm allowed us to compare how suited different populations of sONα RGCs are to encode the presence of a cricket. Each encoder consisted of two populations of BCs, one with weak (w) and one with strong surround suppression (s) with parameters based on the glutamate clusters G1 and G3, respectively (see Materials and Methods), and an RGC population (Fig. 7B). For simplicity, we omitted cluster G2 in the models. The two BC populations were modeled as a square grid of BCs, each with a spatial RF (2D convolution of the input), a nonlinearity (generalized sigmoid) and additive Gaussian noise (Fig. 7, B and C). The RGC population was modeled as a square grid of RGCs, with dendritic arbors (2D convolutions of the input) and distances dependent on the RGC population (Fig. 7, B and C). The decoder was a simple convolution neural network that we trained for each encoder independently.

We created different encoder models based on the functional data described above and anatomical data from (*27*) and (*22*) (see Materials and Methods). The temporal RGC population (*t*_mi_) we modeled received mixed inputs from both BC populations (Fig. 7C) as observed (see Fig. 6L). For this population, the cricket was clearly visible in the population encoding, especially if the cricket was very close (Fig. 7D), which is reflected in the high decoder performance for crickets closer than 20 cm (Fig. 7E). To test the role of the inhibitory surround, we compared the *t*_mi_ RGC population to a population receiving only strong surround (*t*_si_) or only weak surround input (*t*_wi_) (Fig. 7, C to E). The decoder performed best for the temporal population with strong surround inputs only, with significantly higher accuracies than the model with mixed inputs (Fig. 7E), with high performance for close cricket, and a sharp decay in accuracy at around ≈20 cm, similar to the mixed inputs model. Without inputs from BCs with strong surround, the decoder performance was significantly worse (Fig. 7E).

Last, we compared the temporal RGCs to a model of nasal RGCs, with larger dendritic arbors, i.e., pooling inputs from more BCs, larger distances between RGCs, and with inputs from BCs with weak surround only (see Fig. 6L). For this population, the decoder performance was very similar to the performance of the temporal population with weak surround only, indicating only a minor effect of dendritic size and spacing on the observed cricket detection performance. Together, our results suggest that signal integration at the level of temporal sONα dendrites together with changes in the presynaptic circuits indeed are tuned for detecting small objects such as moving insects and, hence, could improve visual hunting performance in mice.

## DISCUSSION

In this study, we investigated the function of the previously reported higher density of sONα cells in the temporal mouse retina (*22*). Specifically, we asked whether this anatomical adaptation goes beyond higher spatial sampling (i.e., higher cell density and smaller dendritic arbors) and is accompanied by distinct functional changes. To this end, we looked into the dendritic signal processing of sONα RGCs in different regions of the animal’s retina.

We found that dendritic Ca^2+^ signals in nasal sONα RGCs were mostly ON sustained with modest surround suppression, as it was reported for this cell type in earlier studies (*21*, *27*, *28*). In contrast, temporal sONα RGCs additionally exhibited dendritic Ca^2+^ signals with strong surround suppression in more than a third of the dendritic segments we measured from. This strong surround suppression was already present in the excitatory synaptic inputs onto these RGCs, pointing to the involvement of presynaptic mechanisms.

Using computational population models of these cells, we analyzed how nasal and temporal sONα RGCs encode movies of small moving objects such as crickets. Our modeling results indicate that the observed differences in synaptic inputs could be a regional adaptation beneficial for tasks like visually guided hunting.

### Regional adaptations in the retina

Regional cell type–specific adaptations [reviewed in (*1*, *29*)] have long been studied in the retina. For example, (*30*) observed in cats that some RGC types become denser while significantly decreasing in dendritic field size toward the central retina, with the highest densities and smallest dendritic arbors in the area centralis. Such regional changes in cell density/dendritic arbor size are common in vertebrates and typically linked to visual acuity—the denser the mosaic of a cell type, the higher its spatial resolution. Regional adaptations also occur upstream of the RGCs; for instance, cone photoreceptors (cones) in the primate fovea are slower than peripheral cones and, hence, shape foveal perception (*31*).

In mice, it has been observed that a large fraction of eye movements are compensatory and counteract their head/body motion (*32*). Therefore, it is expected that mice stabilize the visual scene on their retina with respect to the cardinal axes of the world. As a result, prominent scene features, such as the horizon, tend to fall on distinct parts of the mouse retina, which, in turn, enables its partitioning into specialized regions. This is different from, for example, primates, which also feature a specialized region—the fovea—but use it to “scan” the visual world (*33*, *34*). It has, therefore, been proposed that the ventral mouse retina, which is “looking up” and covering the upper visual field, may be specifically tuned for detecting birds of prey in the sky [reviewed in (*1*, *29*)].

Recent mouse studies have revealed several regional adaptations at all retinal levels, including a prominent opsin expression gradient along the dorsal-ventral axis (*7*–*10*), region-dependent axonal territory sizes in OFF BCs (*35*), and an overall lower RGC density in the dorsal retina (*16*, *17*). There were also several RGC type–specific regional adaptations reported, such as distinct density distributions (*17*, *19*, *22*, *36*), at times associated by changes in morphology (*18*, *22*, *35*).

Adaptations on the functional level have only been reported for a few RGC types so far, for instance, for the transient OFF alpha cells (tOFFα) (*21*) and the JAM-B cells (*18*), both of which vary in their response along the dorso-ventral axis. tOFFα cells were reported to feature more sustained light responses in the dorsal versus the ventral retina (*37*), while JAM-B cells change from being (modestly) direction-selective in the dorsal to color-opponent in the ventral retina (*38*, *39*). In the latter, the functional change is accompanied by a change in dendritic arbor morphology (*18*). In the present study, we have identified another RGC type, the sONα, that exhibits fine-grained regional functional differences—supporting the view that local adaptations of functional properties and, hence, distinct roles in different regions of the retina, are not the exception but the rule in animals like mice. Such functional regionalization further adds to the already astonishing diversity of RGC signals (*28*, *40*) in the mouse retina.

### Functional properties of sONα cells

The sONα RGC can be distinguished relatively easily from other RGC types based on their highly sustained somatic ON responses, large soma sizes, and SMI-32 immunoreactivity (*20*, *21*, *23*, *41*). For this reason, they are ideally suited to investigate regional adaptations of retinal circuits.

sONα RGCs have a high base firing rate under steady illumination (*20*, *21*, *42*) driven by excitatory synaptic inputs (*20*) originating mostly in type 6 and type 7 BCs (*26*, *27*, *43*). While sONα cells express low levels of melanopsin and are, hence, intrinsically photosensitive (*44*), their light response is dominated by the synaptic inputs (*41*). The surround RF of sONα is antagonistic (*20*, *41*), presumably by suppression of excitatory presynaptic inputs (*20*, *26*).

Previous studies have already shown the systematic variation in sONα RGCs morphology (*22*, *23*), with a temporal hotspot where the cells have the highest density and the smallest dendritic fields. Bleckert *et al.* (*22*) also showed that the temporal cells have a higher coverage factor than nasal sONα cells, potentially increasing the spatial acuity of the cell population. In this study, we found that nasal and temporal sONα cells exhibit distinct dendritic signal processing, most prominently visible in the surround strengths measured in their dendritic glutamatergic inputs, postsynaptic dendritic signals, and their somatic outputs. This surround suppression was stronger in presynaptic signals compared to postsynaptic Ca^2+^ signals, especially at, or very close to, the soma. This suggests that the surround suppression inherited from the inputs is attenuated at the level of somatic signal integration.

Very recently, Hsiang *et al.* (*45*) have shown that type 7 BCs have a very strong surround, with the center and surround responses virtually canceling each other for spots (bright; flashed for 1.5 s) of a diameter between 200 and 400 μm and completely sign-inverted responses for spots larger than 600 μm, consistent with our glutamate clusters G2 and G3 (Fig. 4, H and I). For type 6 BCs, they found that the strongest response is achieved for spot sizes of around 100 μm, while spots larger than 400 μm resulted in substantially weaker ON responses to the light increment and a below baseline suppression for the light decrement, likely corresponding to our glutamate cluster G1 (Fig. 4, H and I). Hence, our results suggest that temporal sONα RGCs receive more synaptic input from type 7 versus type 6 BCs compared to nasal sONα cells.

In another recent study, Swygart *et al.* (*26*) performed similar experiments to ours, where they also recorded from sONα cells in different regions of the retina. In contrast to our results, they found little surround suppression and no significant difference between different locations of the retina. Also, Johnson *et al.* (*24*), who recorded from temporal sONα cells, reported only weak surround suppression. Two factors may explain these seemingly inconsistent findings.

First, the recordings, for which Swygart *et al*. (*26*) reported the retinal regions, were performed under scotopic conditions [background: ≈0.3 photoisomerization (P*) rod^−1^ s^−1^]; Johnson *et al*. (*24*) recorded under low photopic conditions (background: ≈ 3000 P* rod^−1^ s^−1^). We recorded at photopic light levels (background: ≈10,000 P* cone^−1^ s^−1^ corresponding to roughly 30,000 P* cone^−1^ s^−1^ assuming a collecting area for rods and cones of 0.2 and 0.6 μm^2^, respectively). Second, Swygart *et al.* (*26*) exclusively used a blue light-emitting diode (LED, 450 nm), which drives mostly rods and M cones, while Johnson *et al.* (*24*) used a UV (385 nm) LED that predominantly drives S cones. We used both a UV (390 nm) and a green (575 nm) LED to stimulate both S and M cones equally, as well as rods.

Therefore, these studies investigated different light adaptation levels and spectral composition of the stimuli and, hence, cannot be compared directly. A color dependency of the surround strength seems plausible given the diverse and location-dependent color preference of sONα cells’ surround reported along the dorsal-ventral axis (*14*). However, our data suggest that the main reason for the differences in surround suppression arises from different light adaption levels, as we saw surround inhibition for both combined and separate S- and M-cone stimulation. This is consistent with work studying the surrounds of AII amacrine cells and their contribution to the RFs of sONα cells (*46*). They showed that, at photopic light levels, the AII amacrine cell network gives rise to surround suppression in small (hence, presumably temporal) sONα cells.

The surround suppression we observed could be a means to decrease the overall excitation of temporal sONα cells for bright light levels. Berry *et al.* (*47*) showed that, under photopic conditions and full-field stimulation, intrinsically photosensitive RGCs, including clusters that likely correspond to sONα RGCs, encode visual information relatively poorly compared to mesopic conditions. Especially if the surround is mostly activated during higher light levels, the suppressed surround could counteract over-excitation and stabilize the response of temporal sONα for different light levels.

The results reported in (*26*) may also provide another mechanism for the surround suppression in synaptic inputs and postsynaptic Ca^2+^ signals we observed. Their data suggest that type 6 BCs have multiplexed outputs, with ribbon synapses featuring weak and strong surround suppression, mediated by amacrine cells in individual BCs. While they use this to explain the stronger surround suppression in PixON (EyeWire: 9n) RGCs (*48*) compared to sONα RGCs, this may also be the mechanism that enables increasing dendritic surround suppression in temporal sONα cells with minimal changes in the circuit. Therefore, rather than adjusting the ratio of their presynaptic partners to receive stronger surround suppression, temporal sONα cells may instead connect to different ribbons within the same type 6 BCs.

We also found differences in transience that were not only dependent on retinal region and dendritic distance to soma but also on the stimulus with no simple relationship between pre- and postsynaptic signals. Especially for the presynaptic signals, the transience was very different for spatially uniform versus nonuniform stimuli. This stimulus dependence may arise from inputs onto BC terminals of large GABAergic amacrine cells that are more strongly activated by uniform stimuli and, therefore, provide stronger inhibitory feedback. In ON cone BCs, this inhibition is predominantly mediated by γ-aminobutyric acid type A receptors (*49*, *50*), which provide fast transient inhibition (*51*). Consequently, glutamate release may become less transient for spatially more uniform stimuli, especially in BCs with strong GABAergic inputs. Similarly, this might also explain the difference in pre- and postsynaptic temporal RF kernels, as postsynaptic RFs are larger and integrate signals from multiple BCs.

### A role for sONα RGCs in visually guided hunting

Several studies have shown that mice use their vision to hunt prey (*24*, *25*, *52*–*54*). In particular, the binocular region in the temporal mouse retina—where sONα cells have their highest density—seems to play a critical role (*24*, *25*). Johnson *et al.* (*24*) showed that of the 40+ RGC types in mice, only a subset of 9 types make ipsilateral connections to the brain. Moreover, the authors showed that from these nine types, only five, including the sONα RGC, have reliable responses to a stimulus mimicking a moving insect, suggesting that these RGC types are critical for successful hunting. In a related study, Holmgren *et al.* (*25*) showed that mice bring the image of their prey on a relatively small spot in the temporal retina with high accuracy, coinciding with the region where sONα RGCs have the highest density. Together, this suggests that sONα RGCs in this temporal high-density region play a role in hunting and that any regional functional adaptations relate to this and related behavioral tasks. In our study, we provide further evidence that temporal sONα RGCs play a dedicated role in visually guided prey capture by demonstrating that the regional adaptations we found in these cells can indeed be advantageous for such a task.

We do not propose that hunting behavior is supported by a single RGC type; certainly, other RGCs also play vital roles in such a complex behavioral task. For example, OFF RGCs, in particular, would be well suited to complement the signals from sONα RGCs. Among the nine ipsilaterally projecting RGCs, there are only two OFF types: sOFFα (EyeWire: 1wt) and tOFF (EyeWire: 4i) (*24*), with the sOFFα being especially interesting because its highest density region coincides with that of sONα (*22*).

Beyond the retina, Krizan *et al.* (*55*) recently showed that narrow-field neurons in the superior colliculus of mice are used for hunting and that these neurons receive input from both direction and nondirection selective RGCs. Eliminating retinal direction selectivity did not affect the animals’ hunting behavior and success, suggesting that nondirection selective RGCs, therefore potentially sONα RGCs which also project to the superior colliculus (*44*, *56*, *57*), provide critical input for prey capture to these neurons.

### Linking the retina to behavior

In this study, we used an encoder-decoder paradigm that allowed us to analyze the effects of different inputs and dendritic field sizes of sONα RGCs systematically and efficiently. The assumptions of the encoder, i.e., the sONα population model, were rather conservative and reduced to the main regional differences we observed, namely, the morphological differences and the different levels of surround suppression in the presynaptic input.

For the sake of simplicity, we excluded other factors that differ between the populations, such as temporal effects, inhomogeneities (e.g., non-Gaussianity) in the spatial structure (*27*) of the RFs, and other RGC types. Therefore, our encoder-decoder model may well underestimate the performance of the biological counterpart. In a future study, it would be interesting to see how the model’s performance in the encoding-decoding task changes when including the signals of any of the aforementioned OFF types. Another limitation of our model may come from the videos we used. While they were reconstructed from freely moving mice hunting crickets and thus provided an accurate visual input as seen by these mice, the videos were recorded in an artificial, well-lit environment. Hence, the visual scene statics, in particular, concerning background and illumination [“backdrop,” see (*58*)], were far from naturalistic.

In general, it is difficult to relate the retinal output to something as complex as behavior. To address this problem, there are several approaches in the literature that can be broadly grouped as follows. In some studies, the researchers looked at behavioral data and tried to link their observations to previous findings about the retina [e.g. (*25*, *59*)]. Other studies used functional recordings in response to either artificial or natural stimuli and tried to draw conclusions about the behavioral relevance [e.g. (*19*, *60*)]. The aforementioned study by Johnson *et al.* (*24*) is particularly interesting in this respect because the authors used both behavioral data and functional retinal recordings to find the most important RGC types for this task. With the encoder-decoder paradigm used in this study, we were able to make another link from functional retinal recordings to hunting behavior. We believe that this approach of combining natural scenes, a retinal encoder model, and a decoder trained on a simple task with direct behavioral relevance offers yet another angle to address the crucial question of functional relevance.

## MATERIALS AND METHODS

### Animals and preparation

Mice used in this study were purchased from the Jackson Laboratory and housed under a standard 12-hour day/night cycle with 22°C, 55% humidity. Mice aged 5 to 15 weeks of either sex were used for all experiments. For the dendritic and somatic Ca^2+^ recordings, we used the wild-type line (C57BL/6J, JAX, 000664; *n* = 13 animals for dendritic recordings and *n* = 5 for somatic recordings). For the glutamate recordings, we used the crossed B6;129S6*-Chattm2(cre)Lowl*/J (ChAT:Cre, JAX, 006410) × B6.Cg-*Gt(ROSA)26Sortm9(CAG-tdTomato)Hze*/J (Ai9tdTomato, JAX, 07909) mouse line that we virus-injected intravitreally to express iGluSnFR in the retina (*n* = 5 animals; see virus injection). All animal procedures were approved by the governmental review board (protocol numbers: CIN 3/18 G, CIN 3/21 M, animal protocol from 31.10.2016, Regierungspräsidium Tübingen, Baden-Württemberg, Konrad-Adenauer-Str. 20, 72072 Tübingen, Germany) and performed according to the laws governing animal experimentation issued by the German government.

The mice were dark-adapted ≥2 hours before tissue preparation, then anesthetized with isoflurane (Baxter, Hechingen Germany), and killed with cervical dislocation. We marked the dorsal side of each eye with dye before quickly enucleating them in carboxygenated (95% O_2_, 5% CO_2_) artificial cerebral spinal fluid (ACSF) solution containing the following (in mM): 125 NaCl, 2.5 KCl, 2 CaCl_2_, 1 MgCl_2_, 1.25 NaH_2_PO_4_, 26 NaHCO_3_, 20 glucose, and 0.5 l-glutamine (pH 7.4). After removing the cornea, sclera, and vitreous body, the retina was flattened on an Anodisc (0.2-μm pore size, GE Healthcare, Pittsburgh, PA) with the ganglion cell side facing up and then transferred to the recording chamber of the microscope, where it was continuously perfused with carboxygenated ACSF (at 35°C and 4 ml min^−^1). All experimental procedures were carried out under very dim red light.

### Virus injection

Before virus injection, the mice (5 to 7 weeks) were anesthetized with 10% ketamine (Bela-Pharm GmbH, Germany) and 2% xylazine (Rompun, Bayer Vital GmbH, Germany) in 0.9% NaCl (Fresenius, Germany). One microliter of AAV9.hSyn.iGluSnFR.WPRE.SV40 (Penn Vector Core, PA, USA) was loaded into a Hamilton syringe (syringe: 7634-01, needle: 207434, point style 3, length 51 mm, Hamilton Messtechnik GmbH). Then, the syringe was fixed on a micromanipulator (M3301, World Precision Instruments, Germany), and the virus was slowly (1 μl/5 min) injected into the vitreous body. Virus-injected mice were used for recordings after 3 weeks.

### Single-cell microinjection

To visualize blood vessels and avoid them when filling individual RGCs, 5 μl of a 50 mM sulforhodamine-101 (SR-101, Invitrogen/Thermo Fisher Scientific, Driesch, Germany) stock solution was added per liter of ACSF solution. Sharp electrodes for single-cell injection were pulled on a P-1000 micropipette puller (Sutter Instruments, Novato, CA) with resistances ranging between 70 and 130 megohm. For Ca^2+^ indicator loading, OGB-1 (hexapotassium salt; Life Technologies, Darmstadt, Germany; 15 mM in water), a synthetic Ca^2+^ indicator dye with high Ca^2+^ affinity (*K*_d_ = 170 nM; Invitrogen) and comparatively fast kinetics (*61*), was loaded into individual RGCs using the single-pulse function (500 ms, −10 nA) of a MultiClamp 900A amplifier (Axon Instruments/Molecular Devices, Wokingham, UK). For the visualization of single RGC morphologies, while recording iGluSnFR signals, 10 mM of Alexa Fluor 594 (Invitrogen/Thermo Fisher Scientific, Dreieich, Germany) was injected into individual RGCs using the same method for Ca^2+^ indicator loading. To allow the cells to fill and recover, we started recordings 1 hour postinjection.

### Electroporation

For somatic recordings, we electroporated the retina (*62*). To this end, the anodisc was placed between two 4-mm horizontal platinum disk electrodes (CUY700P4E/L, Nepagene/Xceltis). The lower electrode was covered with 15 μl of ACSF, while a 10-μl drop of 5 mM OGB-1 dissolved in ACSF covered the upper electrode and was lowered onto the tissue. Then, nine electrical pulses (≈9.2 V, 100 ms pulse width, at 1 Hz) from a pulse generator/wide-band amplifier combination (TGP110 and WA301, Thurlby handar/Farnell) were applied to introduce the Ca^2+^ indicator into the retinal cells.

### Two-photon imaging and light stimulation

We used a Movable Objective Microscope (MOM)-type two-photon microscope (designed by W. Denk, MPI, Martinsried; purchased from Sutter Instruments/Science Products) as described previously (*63*). Briefly, the system was equipped with a mode-locked Ti:sapphire laser (MaiTai-HP DeepSee, Newport Spectra-Physics, Darmstadt, Germany), green and red fluorescence detection channels for OGB-1/iGluSnFR (HQ 510/84, AHF, Tübingen, Germany) and SR-101/Alexa Fluor 594/tdTomato (HQ 630/60, AHF), and a water immersion objective (W PlanApochromat 20 × /1,0 differential interference contrast M27, Zeiss, Oberkochen, Germany). For all scans, we tuned the laser to 927 nm and used custom-made software (ScanM, by M. Müller, MPI, Martinsried, and T.E.) running under Igor Pro 6.3 for Windows (RRID:SCR_000325; Wavemetrics, Portland, OR). Dendritic Ca^2+^ and glutamate signals were recorded with 64 by 16 pixel image sequences at 31.25 Hz with pixel sizes ranging from 0.45 to 0.74 μm. We acquired high-resolution mythology stacks using 512 by 512 pixel image stacks with 0.8- or 1.0-μm z steps. Somatic Ca^2+^ signals were recorded with either 32 by 32 (15.625 Hz) or 64 by 16 pixel (31.25 Hz) image sequences with pixel-sizes ranging from 0.74 to 1.72 μm.

For the light stimulation, a digital light processing projector (lightcrafter, DPM-E4500-UVBGMKII, EKB Technologies Ltd.) was used to display visual stimuli through the objective onto the retina, whereby the stimulus was focused on the photoreceptor layer (*63*, *64*). The lightcrafter was equipped with a light-guide port to couple in external, band-pass–filtered green and UV LEDs (green: 576 BP 10, F37-576; UV: 387 BP 11, F39-387; both AHF/Chroma). The band-pass filter was used to optimize the spectral separation of mouse M- and S- opsins (390/576 Dualband, F59-003, AHF/Chroma). The LEDs were synchronized with the scan retracing of the microscope and intensity calibrated to range from approximately 0.1 × 10^3^ (black background) to 20.0 × 10^3^ (white full field) P* cone^−1^ s^−1^. Steady illumination of ≈10^4^ P* cone^−1^ s^−1^ was present during the scan recordings due to the two-photon excitation of photopigments (*63*).

The light stimulus was carefully centered before every experiment, ensuring that its center corresponded to the center of the microscope’s scan field. A time marker in the recorded data was used to align the visual stimulus with 2-ms precision. For all experiments, the tissue was kept at a constant mean stimulator intensity level for ≥15 s after the laser scanning started and before light stimuli were presented. Light stimuli were generated and presented using the Python-based software package QDSpy (RRID:SCR_016985). We used five types of light stimuli:

1. Binary dense noise (20 by 15 matrix of 30-μm per pixel; each pixel displayed an independent, balanced random sequence at 5 Hz for 5 min) for spatiotemporal RF mapping. The pixel size was chosen to be slightly smaller than the RF center of single BCs [38 to 68 μm in diameter (*65*)], allowing the estimation of RGC dendritic RFs at single-BC resolution.

2. Full-field (800 μm by 600 μm) chirp, consisting of a bright step and two sinusoidal intensity modulations, one with increasing frequency (0.5 to 8 Hz) and one with increasing contrast. The chirp stimulus was repeated three times.

3. Local chirp; like in #2 but as 300-μm diameter spot.

4. Sine-spot; a sequence of light spots, 60 and 300 μm in diameter, with the intensity following a clipped sine wave {max[0,A sin(π t)], where *A* is the maximum intensity} resulting in 1 s of light followed by a 1-s pause before the next spot. The sine-spot sequence was repeated six times.

5. Color spots; a sequence of spots of different sizes (100, 200, 400, 600, and 1000 μm) in blocks of different wavelengths, namely green (G), UV (V), and white (W; i.e., green and UV), ordered as W-V-G-W-G-V. This color sequence was repeated twice, resulting in four repeats per size and color. For both sequences, the spot sizes were in a different, pseudo-random order. Each spot was flashed for 1 s followed by a 3-s pause. To center the spots on the RF of individual cells, we flashed horizontal and vertical bars and adjusted, if necessary, the stimulus center.

### Reconstruction of cell morphologies and retinal location

Immediately following the recording, we captured the full dendritic structure of the RGC using a high-resolution image stack. Through semiautomatic neurite tracing techniques, we reconstructed the cell skeletons of the documented RGCs. For stratification analysis, we flattened the morphologies as follows. We traced the blood vessels in the superficial and intermediate vascular plexus using neuTube (*66*). We fitted a generalized additive model (GAM) to both plexuses using pyGAM (*67*) and computed the stratification depth of dendrites by computing the shortest paths to the GAM regression surface of the superficial vascular plexus. These depths were normalized using the distance between both plexuses and computed as the median distance between both GAM regression surfaces on a regular grid. All subsequent analyses, including the retrieval of morphological parameters (detailed below), were conducted using custom Python scripts.

After the functional and morphological recordings, we recorded the optic disk and the outline of each retina. For the dendritic Ca^2+^ and glutamate recordings, we used Retistruct (*68*) to reconstruct the positions of cells within the retina from these recordings. In a few cases, retinal outlines were incomplete, e.g., a wing was missing and manually adjusted. To define the orientation of the retina, we marked the dorsal side of the eye before enucleation and used this mark to make a dorsal cut in the retina toward the optic disc. In Retistruct, this cut was then set to be dorsal. For somatic recordings, we simply used the distance to the optic disk as the retinal position. Furthermore, to better align our data with previously published data (*17*, *22*), we corrected for the mean angular displacement of 22.1° between defining dorsal based on dorsal marks versus defining dorsal based on the nasal choroid fissure (*69*).

Recorded dendrites and the respective ROIs (see below) were not necessarily well-aligned with the cell morphology reconstructed later. Hence, we aligned each recording field with the respective morphology as follows: We averaged the recording field over time, normalized this average to be between zero and one (clipping values smaller than the 20th and larger than the 90th percentile), and rotated it to match the orientation (with the angle taken from the MOM setup) in the reconstructed skeletons. Next, we cropped the skeleton to a region of approximately 250 μm by 250 μm around the expected position (taken from the raw MOM-setup position readouts) of the recording field, blurred it using a Gaussian 3D filter, and normalized the result to range between zero and one. We iterated over the z layers of the crop and used the matchTemplate function of opencv-python to evaluate how well the field matched with the crop—measured as the mean squared error (MSE)—for all possible *xy* positions within each layer. To penalize matches far away from the expected position, we added a Euclidean distance term to the MSE loss. Last, we used the *xyz* position with the lowest loss as the field’s position with respect to the morphology. ROIs within the field were then projected to the closest dendritic branch based on their Euclidean distance to the nonblurred skeleton (*70*).

### Immunohistochemistry and confocal microscopy

After single-cell recordings, the retina was removed from the anodisc and mounted on a new filter paper (0.8-μm pore size, Millipore). Then, the retina was fixed using 4% paraformaldehyde in 0.1 M phosphate-buffered saline (PBS) for 20 min at 4°C, washed with 0.1 M PBS (6 × 20 min at 4°C), and blocked with blocking solution [10% normal goat serum (NGS) and 0.3% Triton X-100 in 0.1 M PBS] overnight at 4°C. Afterward, the samples were incubated with primary antibodies (anti–SMI-32, 1:100, BioLegend, USA, #801701, and anti-RBPMS, 1:500, Phosphosolution, USA, #1832-RBPMS) in 0.3% Triton X-100 and 5% NGS in 0.1 M PBS for 3 days at 4°C. The samples were then washed with 0.1 M PBS (6 × 20 min at 4°C) and incubated with secondary antibodies conjugated to Cy3 and Alexa Fluor 488 (1:500, Thermo Fisher Scientific, Germany) in 0.1 M PBS overnight at 4°C. After another washing step (6 × 20 min at 4°C), the retina mounted on filter paper was embedded in Vectashield (Vector Laboratories, USA) on a glass slide and covered with a coverslip. Confocal images were taken using a Leica TCS SP8 confocal microscope equipped with 488 and 552 laser lines. Images were taken with HC PL APO 20×/75 and 40×/1.3 oil objectives. Confocal image stacks were aligned with the 2P image stacks, projected to 2D using weighted z projections and brightness and contrast adjusted using custom Python scripts.

### Software environments

Image extraction and semiautomatic ROI placement (see below) were performed using Igor Pro 6.37. All subsequent steps were performed using custom Python code built around a database implemented using DataJoint (*71*). Package versions for the analysis are listed in table S1 and for the model (see below) in table S2.

### Regions of interest

For each field, ROIs were extracted on the basis of dense noise responses as follows (fig. S9) (*70*). First, we computed the SD of the fluorescence intensity for each pixel over time, generating an SD image of the time-lapsed image stack. Pixels with an SD at least twice the mean SD of the field were considered dendritic pixels. Then, the time traces of the 100 dendritic pixels with the largest SDs were extracted and cross-correlated. Last, we grouped neighboring pixels (within a distance of 3 μm) with ρ > ρ_Threshold_ into one ROI, where ρ_Threshold_ was the mean of the resulting cross-correlation coefficients (ρ). In the case of the iGluSnFR data, we drew a dendrite mask manually based on the dendrite in the red channel before we calculated the SD of the time-lapsed recording image (fig. S9, A to D).

Only pixels in the dendrite mask were used for ROI placement, as described above, and further analysis. For the Ca^2+^ data, we also defined “field ROIs” and “proximal dendrite ROIs”: A field ROI was defined as the combination of all ROIs within a field. A field ROI was also a proximal dendrite ROI if the medium dendritic distance to the soma (see below) of all ROIs within the field was smaller than 50 μm. For cells with multiple proximal dendrite ROIs, we used the one resulting in the largest RF estimate.

### Signal processing

After ROI placement, the respective Ca^2+^ or glutamate traces were extracted. For each ROI, we computed raw traces $r_{\text{raw}}$ as means of all ROI pixels. These raw traces were detrended by subtracting a smoothed version of the respective trace $r_{\text{smooth}}$, computed using a Savitzky-Golay filter (*72*) of third polynomial order, from the raw traces $r_{\text{detrend}}=r_{\text{raw}}-r_{\text{smooth}}$. The window length of this filter was 10 s for the sines-spot stimulus and 60 s otherwise.

Detrended traces were then normalized by subtracting the median baseline signal before stimulus onset at time *t*_0_ and by dividing by the SD of the signal

$$r_{\text{norm}}=\frac{r_{\text{detrend}}-\text{median}\left(r_{\text{detrend}}\left[t<t_{0}\right]\right)}{\sqrt{\mathrm{Var}\left[r_{\text{detrend}}\right]}}$$ (1)

This normalization was done independently for each ROI. We did not compare absolute signal strength across ROIs, fields, or cells because it may be too strongly affected by the exact position of the ROI relative to the focal plane and local variations in indicator loading. Last, normalized responses were averaged $a={\langle r_{\text{norm}}\rangle }_{R}$ over stimulus repetitions *R*.

### RF estimation

We mapped RFs of RGCs using the Python toolbox RFEst (*73*). The binary dense noise stimulus (20 by 15 matrix, 30-μm pixels, balanced random sequence; 5 Hz) was centered on the recording field. Normalized traces $r_{\text{norm}}$ were slightly low-pass filtered $r_{\text{filt}}=\mathrm{LP}\left(r_{\text{norm}}\right)$ using a Butterworth filter (*f*_cutoff_ = 3 Hz for Ca^2+^ and *f*_cutoff_ = 5 Hz for glutamate), which improved the yield of high-quality RF estimates. Last, temporal positive-only gradients were computed for each trace

$$\dot{c}=\text{max}\left(0,{\dot{r}}_{\text{filt}}\right)$$ (2)

The stimulus *X*(*t*) was upsampled to the trace sampling rate of 31.25 Hz.

Spatiotemporal RFs F(x,y,τ) were computed from spline-based linear Gaussian models that were optimized with gradient descent to minimize the following loss

$$L=\frac{1}{T}\int_{t=0}^{T}{\left[\dot{c}\left(t\right)-y_{0}-X\left(t\right)\text{S}\text{b}\right]}^{2}+\beta *{\left\|b\right\|}_{1}$$ (3)

where ***S*** is a cubic regression spline basis, *y*_0_ is the inferred intercept, ***b*** are the inferred RF weights, and β is the weight for the L_1_ penalty on ***b*** to enforce sparsity in the RF. The RF was defined as F(x,y,τ)=Sb, where *x* and *y* are the spatial dimensions and τ is the lag ranging from approximately 1.35 to −0.20 s. ***S*** was defined by the number of knots in space and time (*k*_*x*_, *k*_*y*_, *k*_τ_), corresponding to the dimensions *d* of the spatiotemporal RF (*d*_*x*_, *d*_*y*_, *d*_τ_) = (45, 20, 15). We set (*k*_*x*_, *k*_*y*_, *k*_τ_) = (10, 12, 9) for Ca^2+^ and (*k*_*x*_, *k*_*y*_, *k*_τ_) = (10, 16, 12) for glutamate. Further, we set β = 0.005.

Models were trained for at least 100 steps and a maximum of 2,000 steps. If the loss did not improve for 5 steps, training was stopped, and the parameters resulting in the lowest loss were used as the final model.

We decomposed the RFs into a temporal $F_{t}\left(\tau \right)$ and spatial $F_{s}\left(x,y\right)$ component using singular value decomposition and scaled them such that $\text{max}\left(|F_{t}|\right)=1$ and $\text{max}\left(|F_{s}|\right)=\text{max}\left(|F|\right)$. RF quality was computed as

$${\mathrm{QI}}_{\text{RF}}=1-\frac{\mathrm{Var}\left[F\left(x,y,\tau \right)-F_{t}\left(\tau \right)F_{s}\left(x,y\right)\right]}{\mathrm{Var}\left[F\left(x,y,\tau \right)\right]}$$ (4)

Only RFs with *QI*_RF_ > 0.35 were used for the analysis.

For the temporal RFs, which we also call RF kernels, we computed a transience index (TRi) as

$${\mathrm{TRi}}_{\text{Noise}}=2A_{\text{pre}}/\left(A_{\text{pre}}+A_{\text{main}}\right)$$ (5)

where *A*_main_ and *A*_pre_ are the amplitudes of the main peak (i.e., the peak with the smallest lag) and the peak before that, respectively.

The spatial RFs ***F***_*s*_ were linearly up-sampled by a factor of 5. To estimate the RF center outline and size, we fitted contour lines at levels 0.25, 0.3, and 0.35 using matplotlib.pyplot.contour. If, for all levels, there was at least one contour that covered at least 80% of the area covered by all contour lines, the largest contour line at level 0.25 was used as the RF center outline. Other RF fits were discarded. The RF diameter was defined as the diameter of a circle covering the same area as the RF center outline. An additional outline was drawn around this center outline with a 20-μm distance to define the inner border of the RF surround. The RF surround index (*SI*) was defined as

$${\mathrm{SI}}_{\text{RF}}=\frac{\sum_{x,y}F_{s}^{s}\left(x,y\right)}{\sum_{x,y}|F_{s}\left(x,y\right)|}$$ (6)

where the weights of the spatial RF surround $F_{s}^{s}\left(x,y\right)$ were defined as

$$F_{s}^{s}\left(x,y\right)=\left\{\begin{array}{cc} F_{s}\left(x,y\right) & \text{if}\left(x,y\right)\in \text{RF}\;\text{surround}\\ 0 & \text{otherwise} \end{array}\right.$$ (7)

*SI*_RF_ is therefore a value between −1 and +1 and measures the weight and sign of the surround relative to spatial RF as a whole. In some cases, the reconstructed position of the morphology was not ideally aligned with the recorded field positions. For plotting the ROI RFs on the morphology, we therefore subtracted the median offset, i.e., the offset between field RF center and field center, of all field RFs per cell.

### Other response metrics

For the chirp and sine-spot stimuli, the surround index was defined as

$${\mathrm{SI}}_{\text{Stimulus}}=\text{med}{\left[{\mathrm{RI}}_{w}\right]}_{r}-\text{max}\left(0,\text{med}{\left[{\mathrm{RI}}_{s}\right]}_{r}\right)$$ (8)

where *RI*_w_ and *RI*_s_ are the responses to the light increment of the wider (i.e., the step at 2 s of the global chirp/the large spot of the sine-spot) and smaller spot (the step of the local chirp/the small spot of the sine-spot), respectively, with the median taken over stimulus repetitions *r*.

For the color-spots the surround index was computed for each color independently as

$${\mathrm{SI}}_{\text{CS}}=\text{med}\left[{\mathrm{RI}}_{1000}\right]/\text{max}\left(0,\text{med}{\left[{\mathrm{RI}}_{\text{pref}}\right]}_{r}\right)-1$$ (9)

where *RI*_1000_ and *RI*_pref_ are the responses to the light increment of the maximum spot size and the preferred spot (the spot with the strongest response), respectively, with the median taken over spot repetitions *r*. The light increment response *RI* for each repetition was computed as the mean response over Δ*t*_*r*_ seconds after the light increment minus the local baseline, defined as the median response over Δ*t*_*b*_ seconds before it, with Δ*t*_*r*_ = 2 and Δ*t*_*b*_ = 2 for the chirps, Δ*t*_*r*_ = 1 and Δ*t*_*b*_ = 0.25 for the sine-spot, and Δ*t*_*r*_ = 1 and Δ*t*_*b*_ = 1 for the color spots.

We also computed a transience index for the local chirp, which was defined as

$${\mathrm{TRi}}_{\text{Chirp}}=\text{med}{\left\{\left[RP_{a}-\text{max}\left(0,RP_{b}\right)\right]/\left[RP_{a}+\text{max}\left(0,RP_{b}\right)\right]\right\}}_{r}$$ (10)

where *RP*_a_ and *RP*_b_ are the responses to the light increment defined as the 90th percentile of the response in a 1-s window minus the local baseline (i.e., the median of the 2 s before the light increment), with the median taken over stimulus repetitions *r*. For *RP*_a_, this window started directly at the light increment, and for *RP*_b_, it was the last second of the light step, i.e., starting 2 s after the light increment. If *R*_*a*_ was negative for any repetition, no transience index was computed.

### Quality filtering

To measure response quality for repeated stimuli, we computed a signal-to-noise ratio (SNR) quality index (*28*)

$${\mathrm{QI}}_{\text{SNR}}=\frac{\mathrm{Var}{\left[{\langle C\rangle }_{r}\right]}_{t}}{{\langle \mathrm{Var}{\left[C\right]}_{t}\rangle }_{r}}$$ (11)

where *C* is the *T*-by-*R* response matrix (time samples by stimulus repetitions) and 〈〉_*x*_ and *Var*[]_*x*_ denote the mean and variance across the indicated dimension *x*, respectively. We only included dendritic ROIs that had a good SNR for the local (l) or global (g) chirp

$$\left({\mathrm{QI}}_{\text{SNR}}^{l}\geq 0.35\right)|\left({\mathrm{QI}}_{\text{SNR}}^{g}\geq 0.35\right)$$ (12)

For the somatic recordings, we observed tissue motion in the *z* direction in a few recordings. In these cases, we manually removed individual, affected trials. For the color spots, we measured the response quality as in Eq. 11 for each color independently, using only the responses to spots of 300 and 400 μm. We only included somatic ROIs with a good color spot (cs) and global chirp (g) response

$$\left({\mathrm{QI}}_{\text{SNR}}^{g}\geq 0.5\right)\&\left({\mathrm{QI}}_{\text{SNR}}^{\text{cs}}\geq 0.5\right)$$ (13)

### Functional clustering

We clustered the Ca^2+^ and glutamate datasets independently using the same method. For both datasets, we downsampled averages from local and global chirp responses by averaging the signal over every four consecutive time points, concatenated the local and global chirp, and clustered them using Ward hierarchical clustering, implemented in scikit-learn (*74*). We used a Euclidean distance metric and a threshold of 110 that we selected on the basis of the respective dendrograms, resulting in three clusters for both datasets.

### Morphological metrics

Soma size was defined as the soma area in the image frame where the soma appeared the largest. Dendritic field area was defined as the area spanned by a convex hull around the z-projected skeleton of a cell; the respective diameter was defined as the diameter of a circle with an equivalent area. The dendritic distance to soma for an ROI was defined as the length of the shortest path from an ROI to the soma center along the dendritic arbor and computed with MorphoPy (*75*).

### Statistical analysis

All levels of statistical significance are reported as *P < 0.05, **P <0.01, and ***P < 0.001. We used GAMs to analyze response properties as a function of dendritic distance to soma. GAMs are an extension of GAMs that allow linear predictors to depend on smooth functions of the underlying variables. Here, we used the following GAM model

$$g\left(\mu \right)=\beta_{0}\left(z\right)+f\left(x,z;k\right)+r\left(c\right)$$ (14)

where the outcome variable *y* has expectation μ, *g* is a link function, β_0_ is the intercept per group *z*, *x* is the predictor variable, *f* is a smooth function, and *r* is a random effect per cell *c*. GAMs were implemented in R using the mgcv package. The smooths *f* were penalized cubic regression splines with dimension *k*, where lower values of *k* mean smoother fits. For each fit, we compared different values for *k*, models from the Gaussian or scaled t-distribution family, and models with and without random effect *r*. We selected the best model based on the Bayesian information criterion and diagnostic plots. To compare the differences between groups, we used plot_diff of the itsadug R package while excluding the random effect per cell. For the comparison of two groups, we used 95% confidence intervals (CIs); for three groups, we used $98.\bar{3}\%$ CIs to adjust for multicomparison.

### Cell identification

We identified sONα cells based on combinations of the following features dependent on the dataset (i.e., glutamate, dendritic Ca^2+^, or somatic Ca^2+^): morphology (fig. S1), stratification in the inner plexiform layer (IPL) (fig. S2), soma size and non-round shape (figs. S3 and S8A), and sustained ON responses in the Ca^2+^ signals in proximal dendrites (fig. S4D) or soma (fig. S8B). In two cases, we also did SMI-32 and RBPMS stainings (fig. S10).

The stratification depths and profiles were quite consistent across cells, retinal location, and datasets. However, compared to the morphologies from the EyeWire dataset (*76*), stratification profiles were slightly shifted toward the OFF layer, presumably because of differences in the method for IPL border estimation.

Dendritic field diameters ranged from ≈400 μm in the nasal retina to ≈150 μm in the temporal retina (fig. S1), similar to previous reports (*22*). Cells in the glutamate dataset were slightly larger on average in both regions. It is possible that the dye used in the glutamate experiments allowed to better resolve the finest dendritic tips, resulting in somewhat larger cells. The dye may have also influenced the soma measurements, which were also somewhat larger in the glutamate dataset (fig. S3). Moreover, differences in dendritic field size may also be due to variations in retinal location of the recorded cells for the two datasets.

### Model

We created population models of sONα RGCs. We derived the model parameters from our data and previously published data (see below). To simulate the differences between nasal and temporal sONα RGCs, we used different parameters for dendritic wiring and RGC spacing (see below). We used these models to encode visual scenes from freely moving mice that were hunting crickets in an arena, recorded in (*25*). The visual scenes we used were reconstructions of “eye views,” i.e., projections of the visual scene onto the retina, including the body, head, and eye movement of the mouse.

#### Data

The eye views were generated similarly to (*25*) but with 67 frames per second. To minimize projection artefacts, we rotated the area of interest toward the region of the retina where we recorded our t cells [corresponding to the point (0.63, −0.35) for the left eye and (−0.63, −0.35) for the right eye view in (*25*)] before projecting it into equidistant coordinates. The respective videos were cropped to a total size of 1975 μm by 1975 μm and downsampled to a pixel-size of 5 μm. Furthermore, we used a copy of this dataset but with the cricket removed from the videos. We used a total of 452 videos with a total duration of 1464.3 s equal to 69,008 frames. The data were split into training (78.5%), development (15.2%), and test (6.3%) sets. In the dataset, videos were recorded in pairs for both eyes; when splitting the data, we ensured that all these pairs from individual runs were in the same split. Similarly, frames with the cricket removed were always in the same split as their counterparts with the cricket.

#### Encoder model

We implemented the encoder as a convolutional neural network (CNN) in tensorflow. The CNN consisted of the following layers, resembling the vertical pathway in the retina:

1. Input (output shape: 395 × 395).

2. BC spatial RFs [output shape: 2 × (111 × 111)], implemented as a 2D convolution [kernel size: 65 × 65; stride: 3, equal to a distance of 15 μm (*27*)].

3. BC nonlinearities (output shape as above), implemented as a generalized sigmoid function.

4. BC noise (output shape as above), implemented as additive Gaussian noise with zero mean and an SD σ.

5. RGC dendrites (output shape: *k* × *k*; with *k* = 9 for the nasal population and *k* = 19 for the temporal population), implemented as a 3D convolution [Nasal population, kernel size: 21 × 21; stride: 10, equal to 150 μm; temporal population, kernel size: 27 × 27; stride: 5, equal to 75 μm; (*22*)].

All encoder models we implemented shared the model parameters of the BC layers. Only the last layer, the RGC dendrites layer, differed between different encoder models.

The kernels of the BC spatial RFs layer were derived from the glutamate clusters G1 and G3 (Fig. 6) as follows. A Difference of Gaussians (DoG) was fitted to each spatial RF using the python package Astropy (*77*). During fitting, the mean and covariance matrices of the center and surround Gaussian fits were tied, except for a linear scaling of the covariance matrix. DoG fit quality was computed as

$${\mathrm{QI}}_{\text{DoG}}=1-\frac{\mathrm{Var}\left[F_{s}\left(x,y\right)-F_{\text{DoG}}\left(x,y\right)\right]}{\mathrm{Var}\left[F_{s}\left(x,y\right)\right]}$$ (15)

where $F_{\text{DoG}}\left(x,y\right)$ is the DoG fit. Last, parameters of good fits, i.e., *QI*_DoG_ ≥ 0.35, were averaged over all ROIs per cluster and used as the BC kernels with the maximum amplitude being set to one. As nonlinearities, we used the following sigmoid function

$$f\left(x\right)=\frac{3}{1+29\;\text{exp}\left[-b\left(x-d\right)\right]}$$ (16)

where *x* is the input and *b* and *d* are parameters per population. We defined these parameters such that the output for both groups *i* ∈ {*w*, *s*} of simulated BCs had the same response for the respective mean input ($f\left({\bar{x}}_{i}\right)=0.1$ per frame) and mean plus one SD input ($f\left({\bar{x}}_{i}+\sqrt{\text{Var}\left[x_{i}\right]}\right)\approx 0.5$ per frame).

The SD σ of the additive Gaussian noise was set to 0.1. The RGC dendrites layer was implemented as two identical 2D truncated Gaussian-like functions with different weights, representing the different shares of BCs with weak and strong surround inhibition.

The parameters for the scale and cutoff were estimated as follows: First, we created a hexagonal grid representing the BC axonal terminals with a center-to-center spacing of 16 μm (*27*). Then, for each morphology of n and t cells, we estimated the dendritic length per hexagon, with the central hexagon of the grid being placed on the soma (fig. S11A). To each of these distributions, we fitted a 1D truncated Gaussian-like function (fig. S11B), resulting in parameter estimates for the scale (fig. S11C) and the cutoff per cell (fig. S11D). The respective parameter means for n and t cells (fig. S11, C and D) were then used to construct the 2D RGC dendrites in the model (Fig. 7C).

#### Decoder model, training, and evaluation

For each encoder model, we created a decoder model. The decoders were implemented as ensembles of 10 CNNs with identical architectures. Each CNN consisted of five layers of 2D convolutions (three filters, ReLU activation, zero padding, and L_2_ regularization with ω = 0.001) followed by 2D max-pooling (pool size: 2 × 2, zero padding). After these layers, a dense layer (eight units, ReLU activation, and L_2_ regularization with weight ω = 0.003) and a single unit output layer (sigmoid activation) followed.

CNNs were randomly initialized and then trained using Adam (batch size 16,384) to minimize the binary cross-entropy loss. We used early stopping based on the validation loss that was tracked starting after 200 epochs: If the validation loss did not improve for at least 0.001 over 10 epochs, training was stopped, and the model resulting in the lowest validation loss parameters was restored. To analyze the models’ accuracies as a function of distance to cricket, we used GAMs as described above (see Eq. 14), except that we used Logistic GAMs and no random effects.
