# Supplementary material for: Task-specific regional circuit adaptations in distinct mouse retinal ganglion cells
Source: Sci Adv. 2025 Apr 23;11(17):eadp7075. doi: 10.1126/sciadv.adp7075 (PMC12017306; doi:10.1126/sciadv.adp7075)
Supplement: Supplementary file 1 — Figs. S1 to S11 Tables S1 and S2 [file sciadv.adp7075_sm.pdf]

Science Advances
AAAS

Supplementary Materials for

Task-specific regional circuit adaptations in distinct mouse retinal ganglion cells

Jonathan Oesterle *et al.*

Corresponding author: Thomas Euler, thomas.euler@cin.uni-tuebingen.de

Sci. Adv. **11**, eadp7075 (2025)
DOI: 10.1126/sciadv.adp7075

This PDF file includes:

Figs. S1 to S11
Tables S1 and S2

Supplementary Text

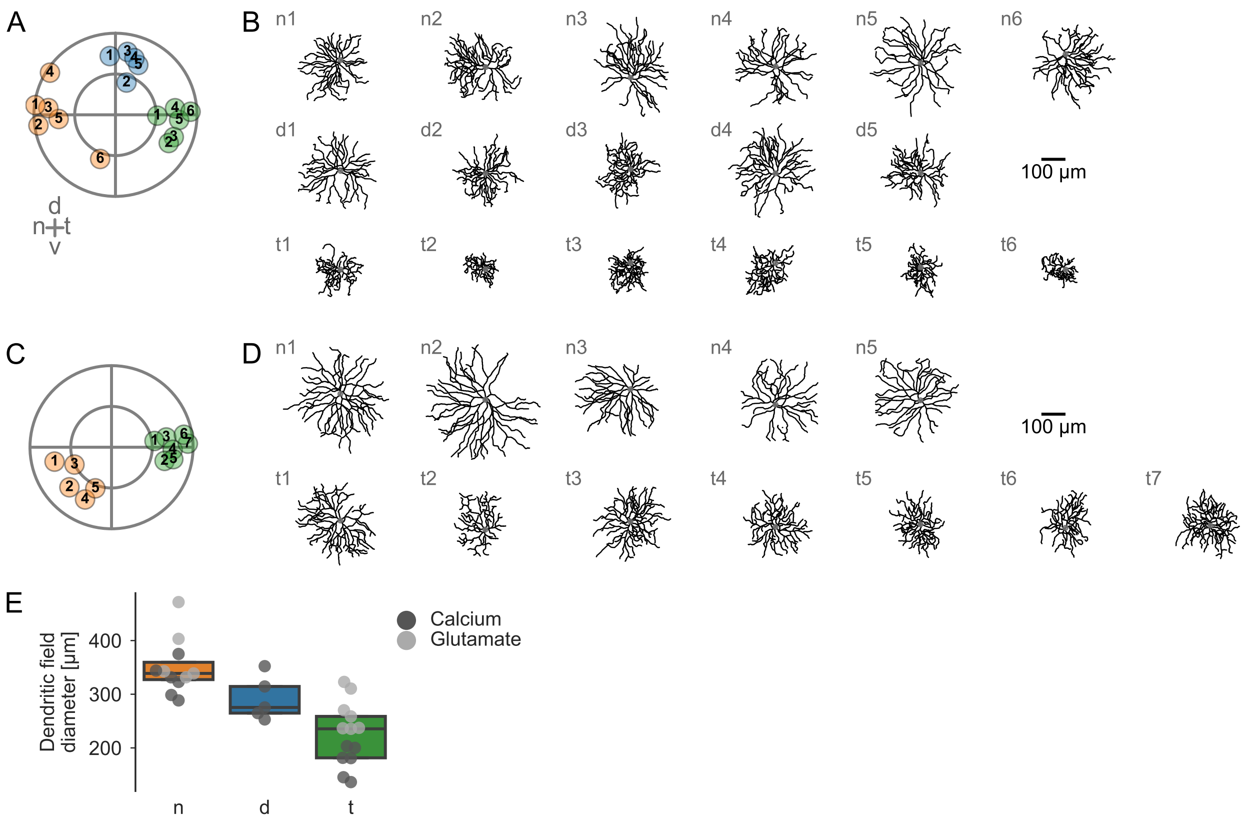

Figure S1: figure

Morphologies and retinal cell locations. (**A**) Cell tags of all cells with dendritic Ca^{2+} recordings (*n*, nasal, orange; *d*, dorsal, blue; *t*, temporal, green). (**B**) Morphologies of cell in (A). Cells are grouped by location: nasal (*n*; *top row*), dorsal (*d*; *middle row*), and temporal (*t*; *bottom row*). Within groups, cells are ordered from most nasal to most temporal. (**C, D**). As in (A, B), but for the glutamate recordings. (**E**) Dendritic field diameter as circle equivalent from dendritic hull of cells sorted by group. Boxes show quartiles. Marker colour indicates if a cell is from Ca^{2+} (dark grey) or glutamate (light grey) dataset.

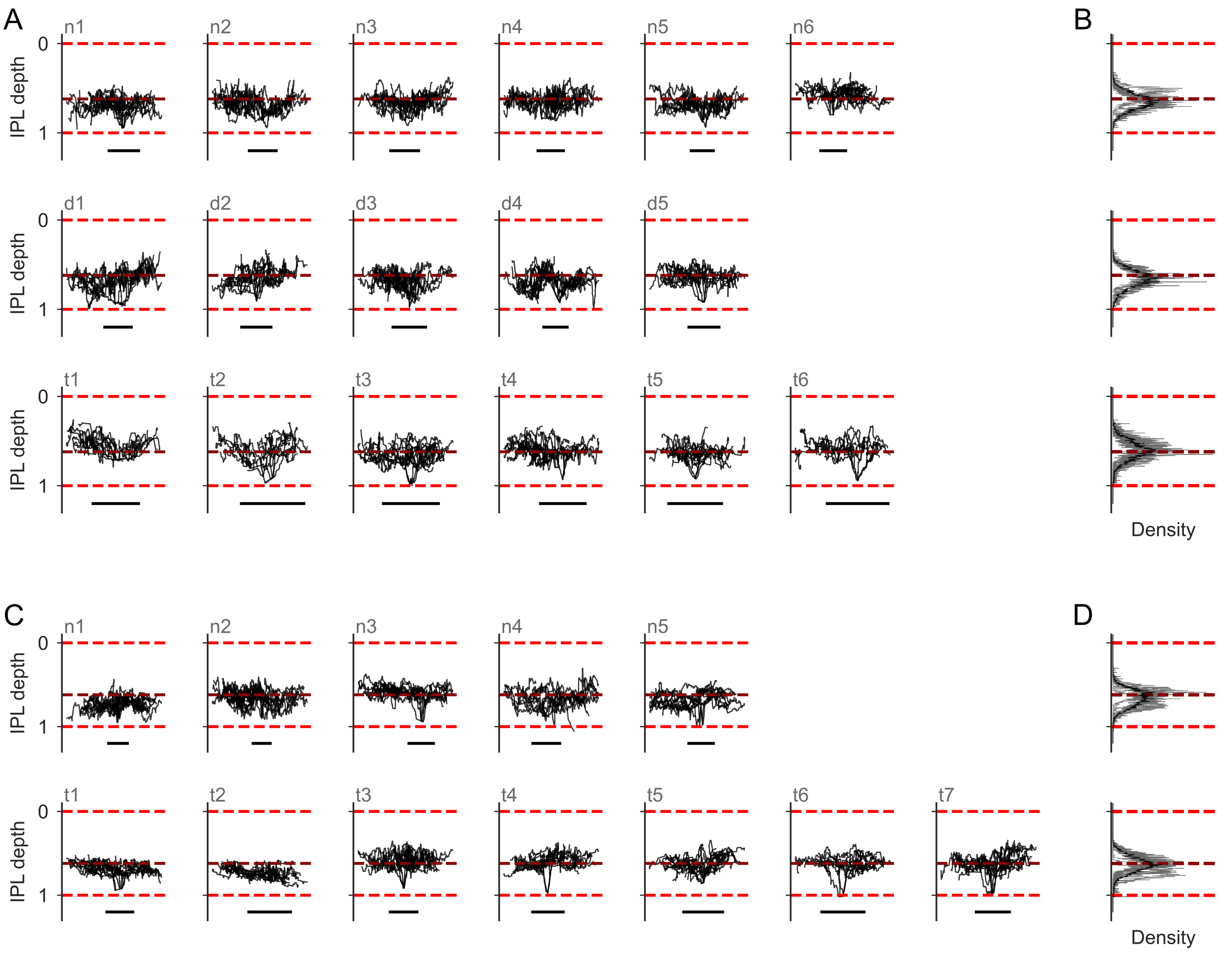

Figure S2: figure

Stratification of cells in the IPL used for identification of sONα cells.. (**A**) Side view of IPL stratification of all cells with dendritic Ca^{2+} recordings. Cells are grouped by location: nasal (*n*; *top row*), dorsal (*d*; *middle row*), and temporal (*t*; *bottom row*). Within groups, cells are ordered from most nasal to most temporal. IPL borders (red) and the ON ChAT band (i.e. IPL depth=0.62) are highlighted. (**B**) Dendritic densities in the IPL for all morphologies (grey) and the mean (black). (**C, D**) As in (A, B), but for the glutamate recordings. (**A, C**) Scale bars indicate 100 µm.

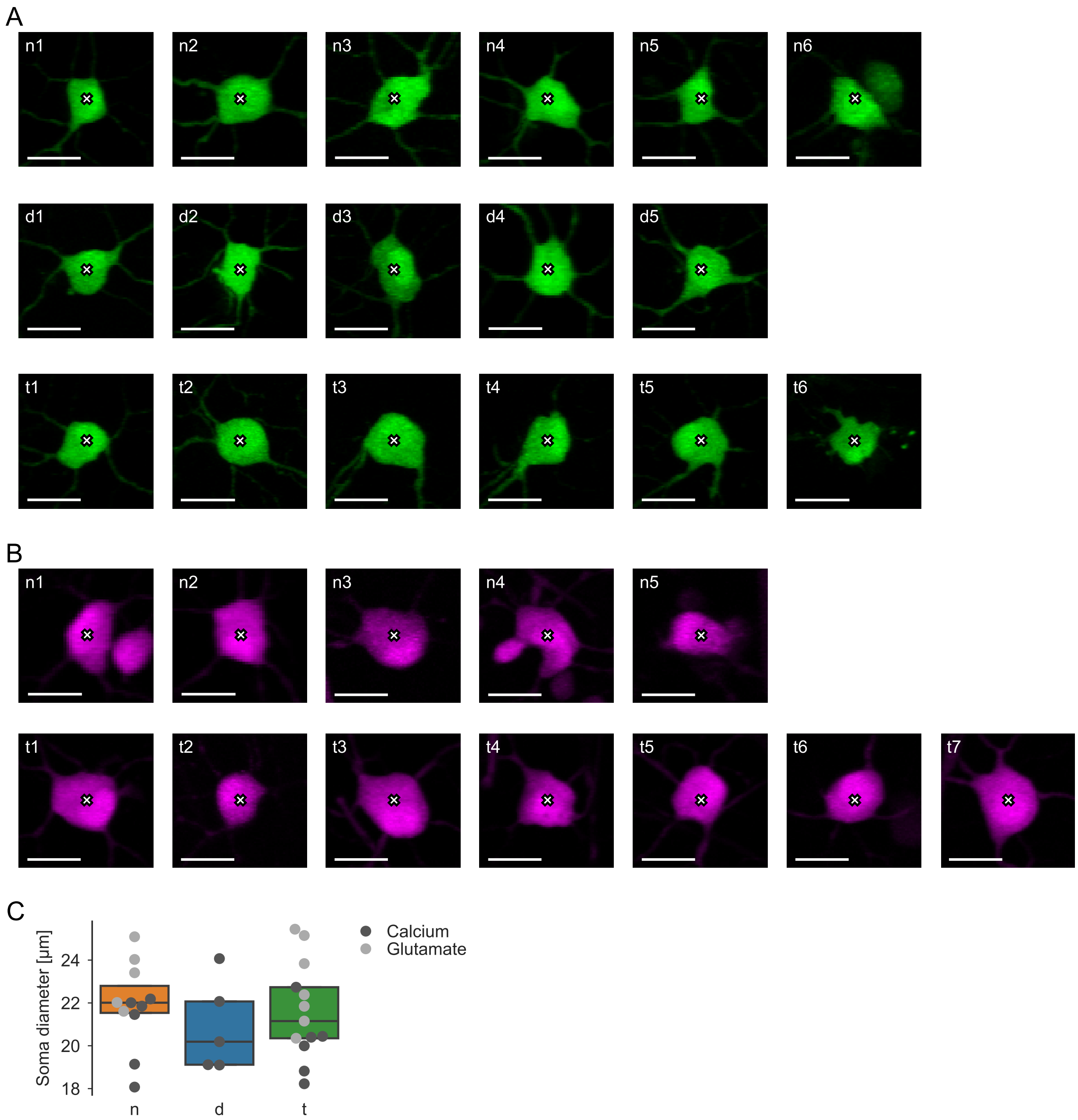

Figure S3: figure

Cell somata used for identification of sONα cells.. (**A**) Maximum z-projection of recorded OGB-1 z-stacks from dendritic Ca^{2+} recordings cropped around the soma. Scale bar indicates 20 µm. Recorded sONα cells are marked (white x). For exact locations, see figure S1A. (**B**) As in (A), but for the Alexa Fluor 594 z-stacks of the glutamate recordings. For exact locations, see figure S1B. (**C**) Soma diameter of cells sorted by group. Boxes show quartiles. Marker colour indicates if a cell is from Ca^{2+} (dark grey) or glutamate (light grey) dataset.

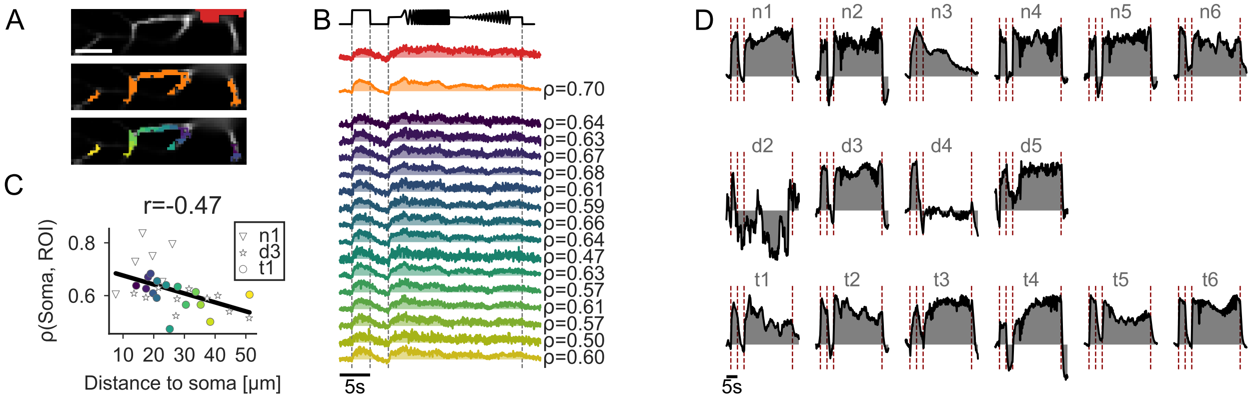

Figure S4: figure

Relation of somatic ROIs, proximal dendrite field ROIs and dendritic ROIs. (**A**) Somatic ROI (*top*), field ROI (*middle*), and dendritic ROIs (*bottom*). The field ROI is defined as the combination of all dendritic ROIs. The somatic ROI was manually drawn for comparison in this field. Scale bar indicates 10 μm. (**B**) Local chirp response averages for all ROIs in (A) (ROIs are colour-coded) and Pearson's correlation coefficient ρ between each ROI's response and the response of the somatic ROI. The shaded area indicates the distance to the baseline (i.e. the median of the first two seconds). All averages were scaled to have the same maximum amplitude relative to the baseline. (**C**) Correlation coefficients from (B) shown as a function of dendritic distance to soma for three cells (see legend; ROIs from A are colour-coded). (**D**) As in (B), but for all proximal dendrite ROIs of the Ca^{2+} data. All cell responses but d2's passed the quality threshold for the local chirp.

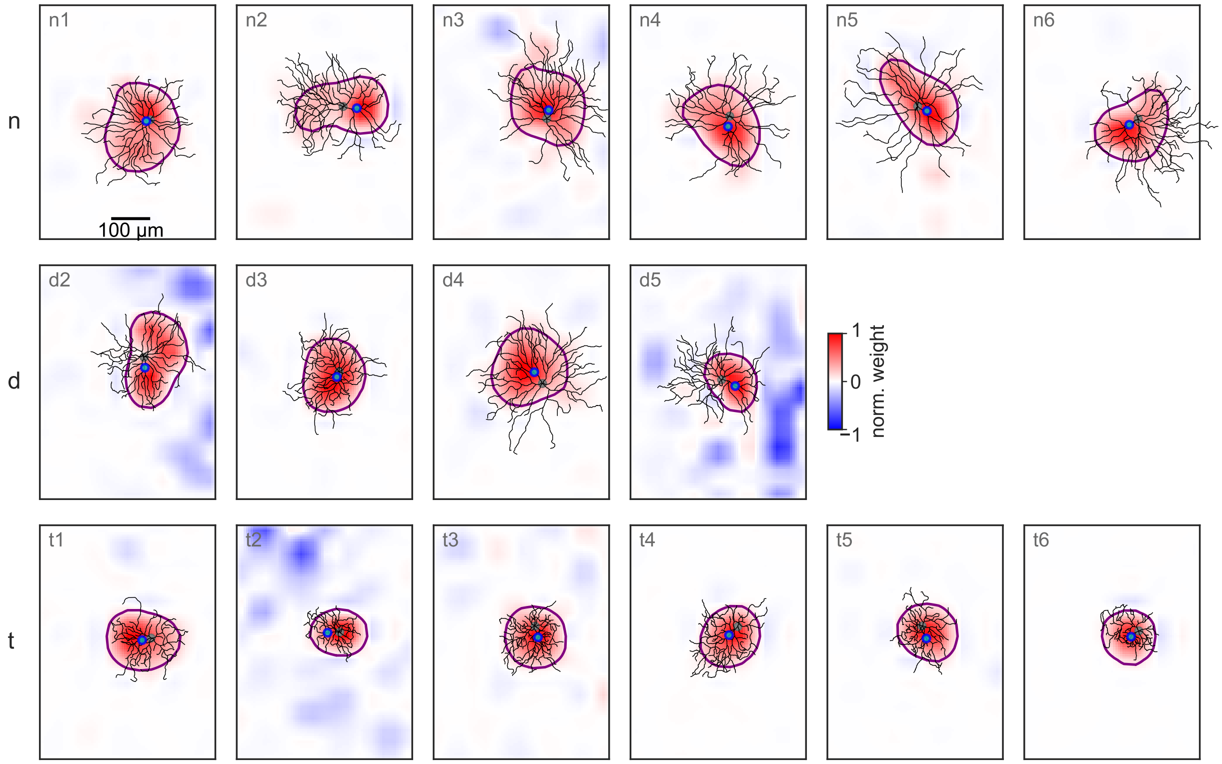

Figure S5: figure

Receptive fields at proximal dendrites. Proximal dendrite RFs grouped by cell location: nasal (*n*; *top row*), dorsal (*d*; *middle row*), and temporal (*t*; *bottom row*). For exact locations, see figure S1A.

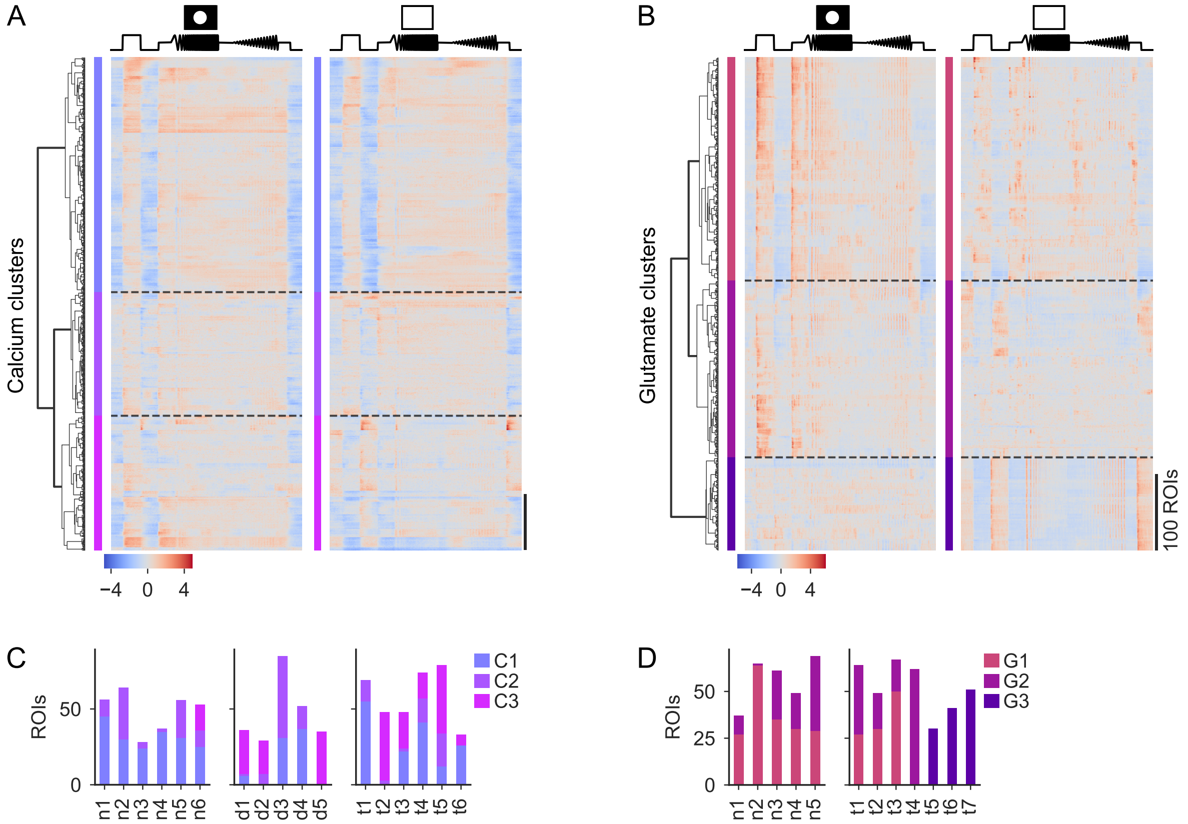

Figure S6: figure

Post- and presynaptic signals in sONα cells can be both clustered into three clusters. (**A**) All chirp responses used for clustering of dendritic Ca^{2+} data and the respective dendrogram. Traces are shown as heat-maps for local (*left*) and global (*right*) chirp. (**B**) As in (A), but for the glutamate data. (**C**) Cluster counts per cell of Ca^{2+} dataset. Cells are grouped by location: nasal (*n*; *left*), dorsal (*d*; *middle*), and temporal (*t*; *right*). For exact locations, see figure S1A. (**D**) As in (C), but for glutamate data. For exact locations, see figure S1C.

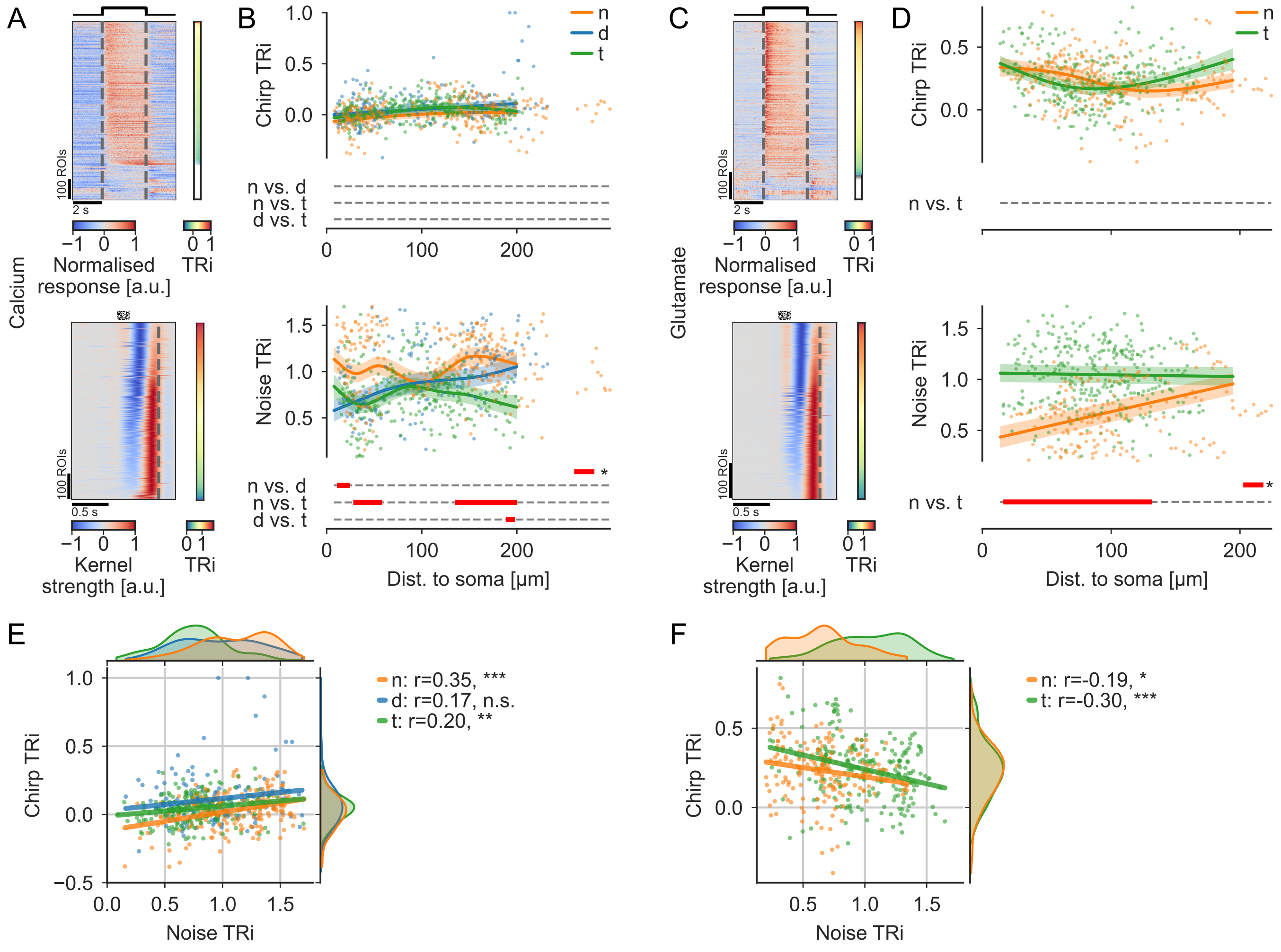

Figure S7: figure

Transience only differs for spatially non-uniform stimuli between retinal regions. (**A, B**) Transience indexes (TRi) from the Ca^{2+} data. (**A**) *Top:* Normalised response averages to the step (i.e. the first seven seconds) of the local chirp stimulus (*left*), ordered by the local chirp transience index (TRi; *right*). Each average was normalised by first subtracting the baseline (i.e. the median of the first 2s) and then dividing by the amplitude (i.e. the maximum absolute value). *Bottom:* Same, but for the temporal RF kernels and the noise TRi. (**B**) Local chirp TRi (*top*) and noise TRi (*bottom*) as a function of ROI dendritic distance to soma by retinal region (colour) and fits from a GAM. (**C, D**) As in (A, B), but for the glutamate data. (**E**) Relationship between chirp TRi and noise TRi by retinal region (colour), fits using linear regression and Pearson's r; correlations are tested for significance per group and adjusted using Bonferroni correction. (**F**) As in (E), but for the glutamate data.

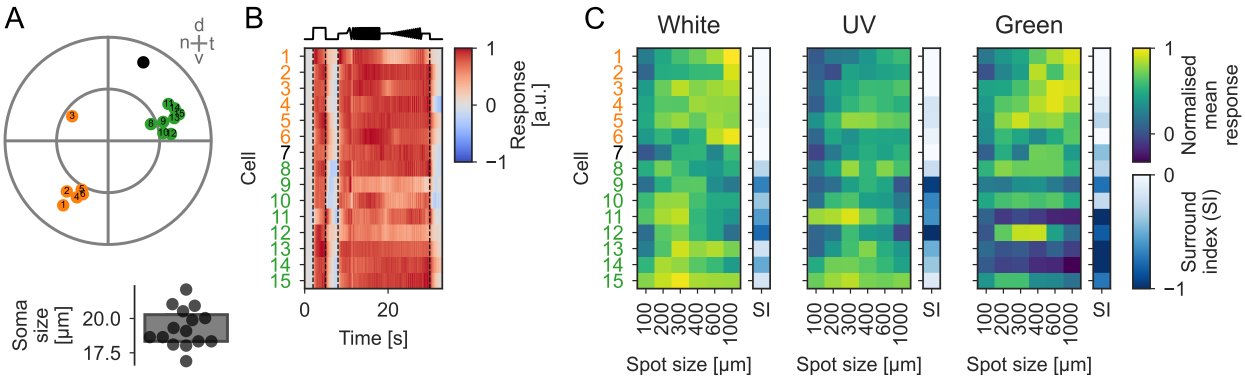

Figure S8: figure

Somatic recordings for chirps and sine-spot stimulus. (**A**) *Top*: Recorded retinal positions. Colours indicate groups as in Fig. 5: nasal (orange), temporal (green), and other (black). *Bottom*: Soma size of cells shown in (A). Box shows quartiles. (**B**) Global chirp responses of cells in (A) as averages over stimulus repetitions. Each average was normalised by subtracting the baseline (i.e. the median of the first 2s) and then dividing by the amplitude (i.e. the maximum absolute value). Note that the normalisation does not allow for a direct comparison of signal strength and, therefore, suppression. We chose this normalisation to highlight the similarity of the relative responses while using the spots to quantify the surround strength in (C). (**C**) Mean responses to the colour spot stimulus, normalised per cell across colours such that the maximum is one, and corresponding surround index for cells in (A) as a function of the spot diameter for white (*left*), UV (*middle*) and green (*right*) spots.

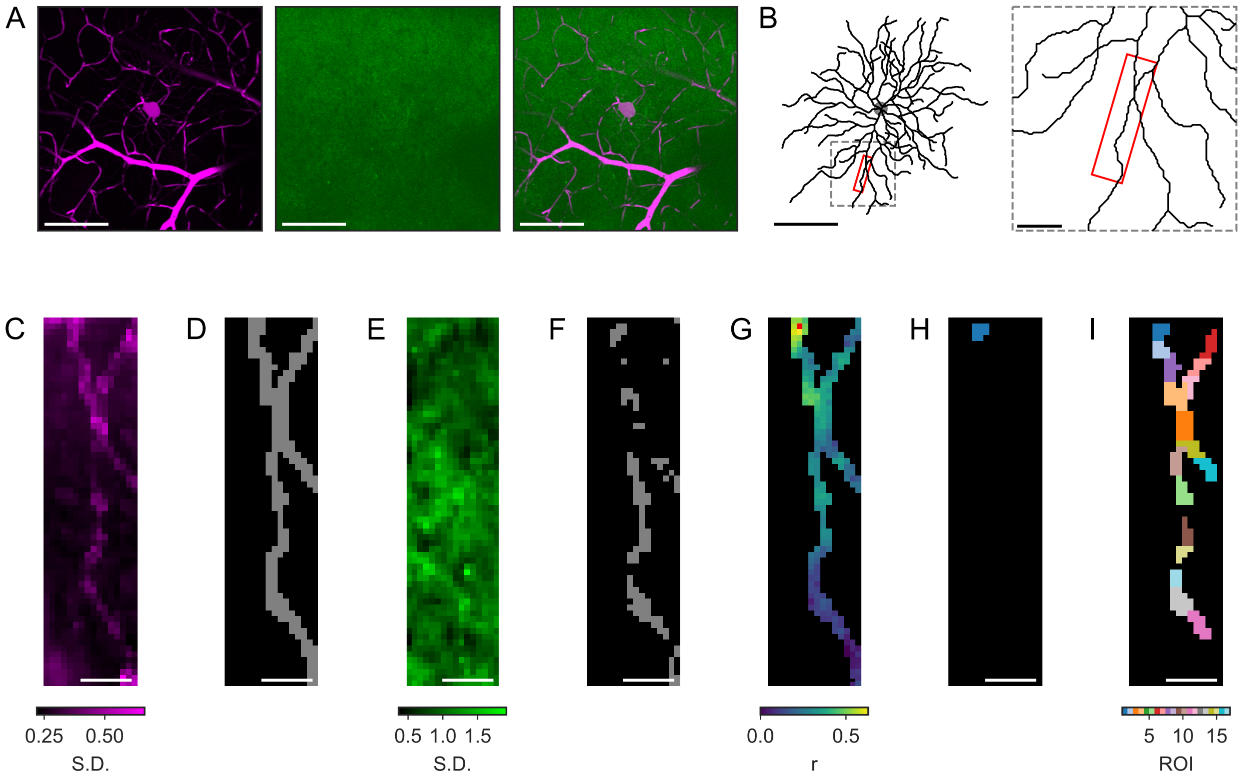

Figure S9: figure

ROI placement of example field from glutamate dataset. (**A**) Weighted z-projection of z-stack from example glutamate cell (t3 in figure S1C, D) for red (SR-101 for blood vessels and Alexa Fluor 594 in injected cell; *left*) and green (iGluSnFR; *middle*) channel, and their overlay (*right*). Scale bar indicates 100 µm. (**B**) Extracted morphology from red channel. *Left*: Full morphology (black), borders of example field (red), and magnified region (grey dashed) that is shown on the *right*. Scale bars indicate 100 µm and 20 µm, respectively. (**C-I**) ROI placement in example field from (B). Scale bars indicate 10 µm. (**C**) S.d. projection over time of red channel. (**D**) Manually drawn dendrite mask to restrict ROI placement. (**E**) S.d. projection over time of green data channel (iGluSnFR). (**F**) 100 brightest pixels (in green channel; (E)) restricted to dendrite mask (D). (**G**) Correlation coefficient r between seed pixel (red) and all other pixels in dendrite mask. (**H**). Single ROI after grouping highly correlated pixels close to the seed pixel. (**I**) All ROIs for this field.

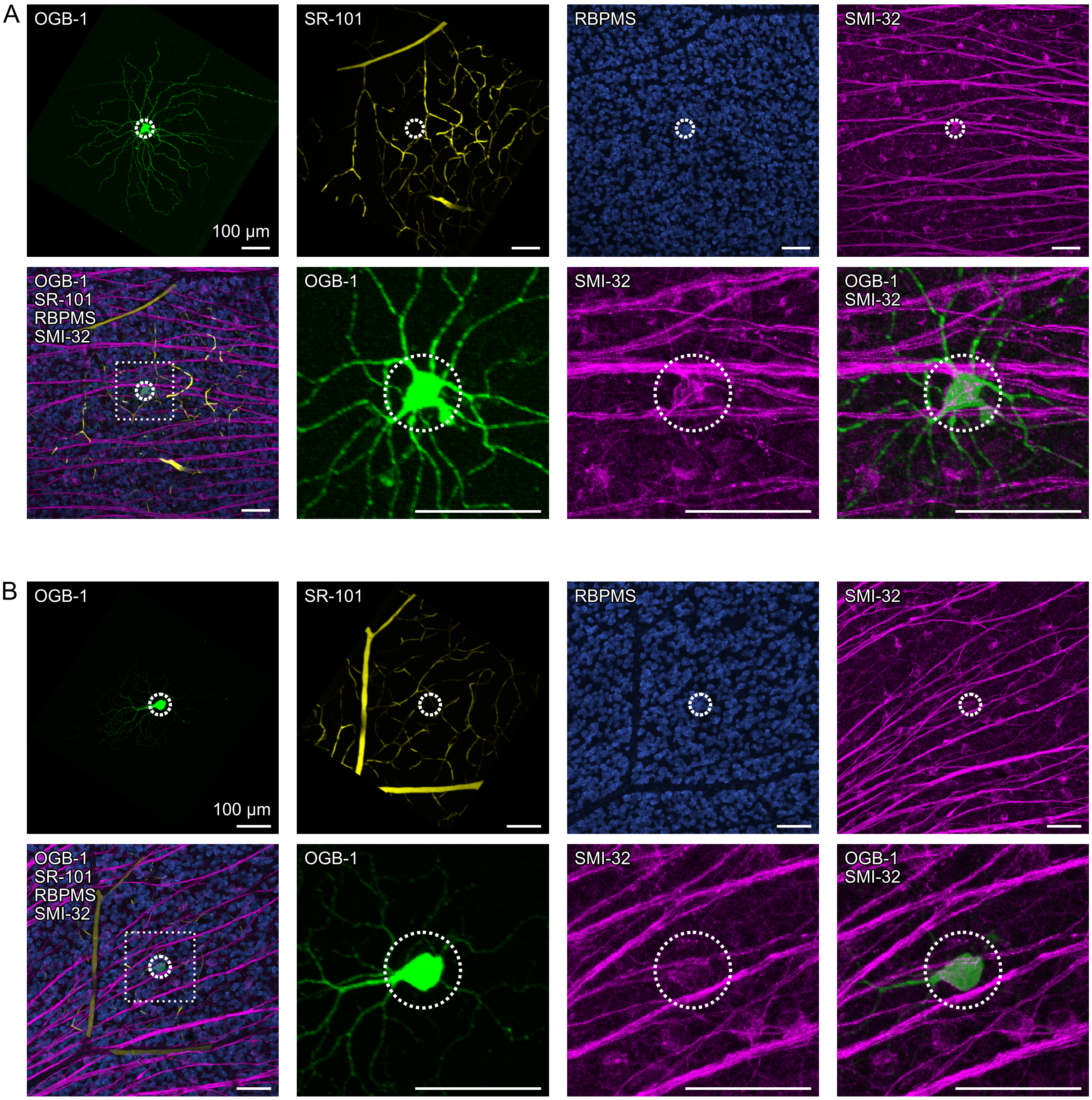

Figure S10: figure

Immunohistochemistry. (**A**) SMI-32 and RBPMS stainings for a nasal sONα RGC (n5 in figure S1A, B). (**B**) As in (A), for a temporal sONα RGC (t4 in figure S1A, B).

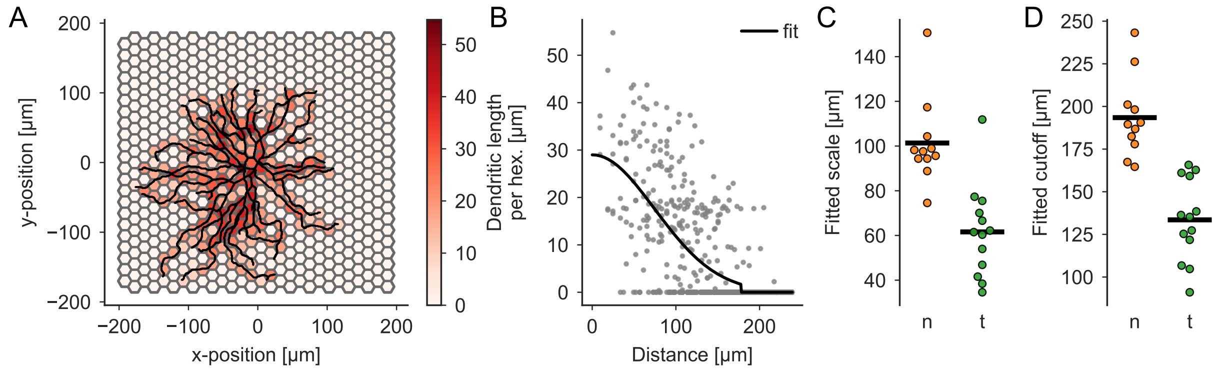

Figure S11: figure

Estimation of dendritic densities for population model. (**A**) Dendritic densities estimated for example cell (n1 in figure S1A, B). The soma is centred on $(0, 0)$. In each hexagon, the dendritic length is estimated (colour-coded). (**B**) Dendritic length as a function of dendritic distance to soma and parametric fit for cell in (A). The fitted function was a truncated bell curve, centred on zero, with an amplitude, scale and cutoff parameter optimised to fit the data. (**C**) Fitted scale parameter for all nasal (*n*; orange) and temporal (*t*; green) cells. (**D**) As in (C), but for the fitted cutoff parameter.

Table S1: Package version for analysis.

Package	Version
Python	3.10.12
datajoint	0.14.1
numpy	1.26.2
pandas	2.2.1
scipy	1.11.4
scikit-learn	1.3.2
pingouin	0.5.4
statsmodels	0.14.1
matplotlib	3.8.2
seaborn	0.13.2
R	4.3.2
mgcv	1.9-0
itsadug	2.4

Table S2: **Package version for encoder-decoder model.**

Package	Version
Python	3.11.5
tensorflow	2.15.0
keras	2.15.0
R	4.2.0
mgcv	1.9-0
itsadug	2.4
